# Adduct annotation in liquid chromatography/high-resolution mass spectrometry to enhance compound identification

**DOI:** 10.1007/s00216-020-03019-3

**Published:** 2020-10-29

**Authors:** Thomas Stricker, Ron Bonner, Frédérique Lisacek, Gérard Hopfgartner

**Affiliations:** 1 Life Sciences Mass Spectrometry, Department of Inorganic and Analytical Chemistry, University of Geneva, 24 Quai Ernest Ansermet, 1211 Geneva 4, Switzerland; 2 Proteome Informatics Group (PIG), Swiss Institute of Bioinformatics and University of Geneva, 7, route de Drize, 1211 Geneva 4, Switzerland; 3 Ron Bonner Consulting, Newmarket, ON L3Y 3C7 Canada

**Keywords:** Electrospray, Metabolomics, Adducts, HRMS, Liquid chromatography, Software

## Abstract

Annotation and interpretation of full scan electrospray mass spectra of metabolites is complicated by the presence of a wide variety of ions. Not only protonated, deprotonated, and neutral loss ions but also sodium, potassium, and ammonium adducts as well as oligomers are frequently observed. This diversity challenges automatic annotation and is often poorly addressed by current annotation tools. In many cases, annotation is integrated in metabolomics workflows and is based on specific chromatographic peak-picking tools. We introduce mzAdan, a nonchromatography-based multipurpose standalone application that was developed for the annotation and exploration of convolved high-resolution ESI-MS spectra. The tool annotates single or multiple accurate mass spectra using a customizable adduct annotation list and outputs a list of [M+H]^+^ candidates. MzAdan was first tested with a collection of 408 analytes acquired with flow injection analysis. This resulted in 402 correct [M+H]^+^ identifications and, with combinations of sodium, ammonium, and potassium adducts and water and ammonia losses within a tolerance of 10 mmu, explained close to 50% of the total ion current. False positives were monitored with mass accuracy and bias as well as chromatographic behavior which led to the identification of adducts with calcium instead of the expected potassium. MzAdan was then integrated in a workflow with XCMS for the untargeted LC-MS data analysis of a 52 metabolite standard mix and a human urine sample. The results were benchmarked against three other annotation tools, CAMERA, findMAIN, and CliqueMS: findMAIN and mzAdan consistently produced higher numbers of [M+H]^+^ candidates compared with CliqueMS and CAMERA, especially with co-eluting metabolites. Detection of low-intensity ions and correct grouping were found to be essential for annotation performance.

**Electronic supplementary material:**

The online version contains supplementary material available at 10.1007/s00216-020-03019-3.

**Figure:**
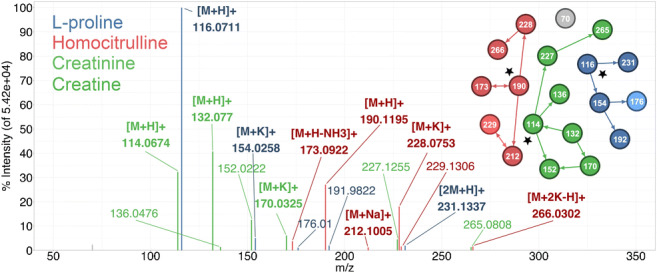
Graphical abstract

## Introduction

Mass spectrometry (MS) and hyphenated liquid chromatography-mass spectrometry (LC-MS) are widely used for qualitative and quantitative analyses in many applications, including metabolomics, pharmaceutical development, forensics, doping control, and proteomics. Ionization of the sample molecules is a critical step and is often achieved using electrospray ionization (ESI) since it is sensitive, straightforward, and amenable to polar molecules. Although analyte ions are frequently formed by the addition or removal of protons to generate [M+H]^+^ ions in positive mode and [M−H]^−^ ions in negative mode, many other ionization processes are known, so even the spectrum of a single analyte may contain many different species [1]. The presence of numerous related species has many consequences. Spectral interpretation is more complicated since the “true” molecular ions, i.e., [M+H]^+^, may be hard to determine or even absent. Data acquisition can be impacted, for example in data-dependent analysis (DDA) where species selected for fragmentation may be redundant and/or refractory, so valuable time is spent collecting useless data. Furthermore, targeted multiple reaction monitoring (MRM) quantitation may seem immune to these effects, but sensitivity can suffer if the signal is distributed between many species. Reproducibility, method development, and transfer may also be affected if the observed species change in unpredictable, compound-dependent ways. Finally, the analysis of more complex samples by LC-MS can produce thousands of features, represented as pairs of a retention time (RT) and a mass/charge value (*m*/*z*), that in fact correspond to a much smaller number of actual analytes [1]. Thus, there is a need to analyze individual mass spectra to identify the ions present (annotation) and determine the molecular weights of the underlying analytes.

Automation is essential for the number of spectra collected in large LC-MS–based studies such as in metabolomics but is also valuable in real-time for DDA and to reduce errors in the analysis of individual spectra, e.g., collected by flow injection analysis (FIA). Although there are numerous software tools for chromatographic feature detection, including the freely available XCMS [2], mzMine2 [3], and MS-Dial [4], automatic annotation is still challenging. For example, Li et al. [5] reported that these packages could generate tens of thousands of signals in mixtures of a thousand metabolites, greatly overestimating the number of real metabolites. These methods utilize chromatographic data to group-related spectral peaks prior to annotation and are highly dependent on the peak-picking parameters since missing important low-intensity peaks, or misassigning the groups, will lead to errors. Manual optimization is a time-consuming and complicated iterative process, especially for inexperienced users, so automatic parameter selection tools have been created for XCMS including Isotopologue Parameter Optimization (IPO) [6] and AutoTuner [7].

Numerous “all-in-one” software packages or frameworks have been released to fully exploit MS1 data, including the widely used XCMS–CAMERA [8]. Although continued support has been provided [9], CAMERA’s decade-old approach to feature grouping and annotation has shown limitations for complex sample analysis, which has led to the development of additional software solutions, including FindMAIN [10] and CliqueMS [11]. FindMAIN uses a weighted scoring system to improve the annotation of ambiguous mass spectra, while CliqueMS implements a unique feature grouping algorithm based on a similarity network. More recently, a machine learning approach for predicting in-source patterns, retention time, and intensity using the chemical structure of compounds was published [12]. This tool aims to improve the assignment of MS signals and decrease the false discovery rate but, while promising, showed a major drawback as new models must be trained for different analytical workflows. From the nearly 200 freely available tools used in metabolomics data processing [8], only a few chromatographic peak detection methods have been benchmarked; rigorous evaluation and cross-comparison of current software solutions is still lacking [13–15]. Furthermore, most current solutions are modules integrated into rigid untargeted analysis pipelines or depend on the outputs of specific peak-picking tools. Thus, there is still a need for a flexible and platform-independent tool that could extensively annotate MS data.

Here, we introduce mzAdan, a nonchromatography-based multipurpose desktop application for the annotation and exploration of convoluted high-resolution ESI-MS data from low molecular weight compounds, including metabolites. We validate mzAdan’s annotation approach on hundreds of FIA mass spectra corresponding to 408 analytes and an LC-MS mixture of 52 standard compounds. We then incorporate mzAdan in an untargeted data analysis workflow with XCMS and benchmark its performance against three other annotation tools, CAMERA, findMAIN, and CliqueMS. Additionally, we investigate the influence of several XCMS parameters on the detection of low-abundance features from standard ionization products, examine their impact on the performance of each annotation tool, and investigate their limitations.

## Material and method

### Data acquisition and processing

#### Flow injection analysis dataset (UNIGE-FIA-MS)

The UNIGE-FIA-MS dataset is the basis of a spectral library described in detail [16]. This library is composed of more than 500 high-resolution mass spectra of metabolite standards from the Human Metabolome Database (HMDB). Data were acquired on a quadrupole time-of-flight (TripleTOF 5600, Sciex, Concord, ON) in FIA mode. Of the hundreds of mass spectra produced, 408 contained the standard protonated forms (intensity ≥ 1% and 500 cps) and were selected for further analysis (see Electronic supplementary material (ESM) Table S1).

The raw data were processed in PeakView (v.2.2, Sciex), where full scans were averaged over about 10 spectra (250 ms accumulation time/spectra), background was subtracted, and the peaks were centroided. Every mass spectrum was ultimately exported as a Mascot generic format file (*mgf*) using an in-house PeakView plugin.

#### Liquid chromatography dataset (UNIGE-LC-MS)

The UNIGE-LC-MS mixture consists of 52 metabolite standards (ESM Table S2) from the HMDB. Data were acquired on an UltiMate 3000 RSLC chromatography system (Dionex, Sunnyvale, CA, USA) coupled to a TripleTOF 6600 (Sciex, Concord, ON, Canada) in positive ionization mode SWATH-MS. Mobile phase A was 5 mM ammonium formate in water with pH adjusted to 3.0 by the addition of formic acid, and mobile phase B was 5 mM ammonium formate in methanol. The gradient was 0–1 min 5% B, 1–21 min 5–95% B, and 21–25 min 95% B with a flow rate of 300 μL/min. The injection volume was of 1.6 μL.

The raw data were processed with PeakView (v2.2, Sciex) and extracted ion chromatograms (EIC) generated for the 52 protonated forms. Spectra were averaged over the full width at half maximum (FWHM) of the chromatographic peaks, and background ions were subtracted from regions preceding and following the peak. These spectra were then centroided and exported as individual *mgf* files.

The Analyst raw data file (*wiff*) was additionally processed and converted to the mzXML open data format with MSConvert (v.3.0.18) [17] to allow analysis with XCMS. MS1 data were centroided using the vendor peak-picking algorithm option, and MS2 data were discarded. Lastly, the whole LC-MS run was converted and exported as a single mzXML file.

#### Pooled human urine sample

A pooled human urine sample (collected anonymously) was analyzed on an UltiMate 3000 RSLC chromatography system (Dionex, Sunnyvale, CA, USA) with an Xselect column HSS T3 XP (2.5 μm, 2.1 mm i.d. × 150 mm, Waters, Milford, MA, USA) coupled to a TripleTOF 5600 (Sciex, Concord, ON, Canada) in positive mode SWATH-MS. Mobile phase A was 5 mM ammonium formate in water with pH adjusted to 3.0 by the addition of formic acid, and mobile phase B was methanol. The gradient was 0–1 min 5% B, 1–16 min 5–85% B, and 16–17 min 85% B at a flow rate of 300 μL/min. Prior to analysis, the urine sample was diluted twice with mobile phase A and 5 μL was injected. The column temperature was kept at 40 °C, and the samples were cooled at 6 °C. For MS acquisition, a single TOF MS acquisition from *m*/*z* 100 to 600 was followed by 20 MS/MS experiments with variable Q1 windows from *m*/*z* 50 to 600 with a cycle time of 691 ms. The collision energy spread was set at 40 ± 30 eV, the ion spray voltage was 5000 V, the declustering potential was ± 80 V, and the source temperature was at 450 °C. The curtain gas was set at 25, gas 1 at 30, and gas 2 at 40.

A total of 35 metabolites were identified with high confidence using the MasterView (v.1.1, Sciex) candidate search functionality, with the AMML spectral library described previously [16], and were chosen for further analysis (ESM Table S3). For all of these, the mass error was less than 5 ppm and the combined score (formula finder score, library score, retention time score) above 60. In addition, each compound was found in at least one previous urine analysis, ideally classified in HMDB as a urine metabolite, and had a high-quality MS/MS match, a retention time deviation below 20%, and a signal-to-noise ratio of above 50.

#### MzAdan annotation software

MzAdan is a cross-platform (Windows, MacOS, Linux) Java desktop application for the annotation of high-resolution full scan mass spectra acquired in positive and negative modes but currently focused on singly charged positive ions. The graphical interface allows quick access to fully customizable and well-described parameters, including various filters (*m*/*z*, intensity, and deisotoping) and multiple annotation sets (adducts, neutral losses, isotopes, oligomers). The application uses two main Java libraries: JmzReader (v.1.2.1) [18] for parsing standard open-source mass spectrometry data formats and JgraphT (v.1.2.0, https://jgrapht.org/) for the annotation based on graph data structures. Additionally, several MzAdan classes were inspired by MzJava [19]. MzAdan can process spectral files in the mgf format and can produce three types of outputs: two tables containing annotations and [M+H]^+^ candidates, as well as the graph itself. The software is available online (https://github.com/sib-pig/mzAdan) with a tutorial detailing the tool interface, settings, and outputs.

#### Generation of reference annotations

For reference, mass spectra of the analytes from the UNIGE-LC-MS dataset and pooled urine sample (52 and 35, respectively) were manually annotated using PeakView and a set of 13 annotations, including adducts and neutral losses (ESM Table S4). Only monoisotopic peaks with intensities above 1% and 500 cps were considered.

#### Evaluation of mzAdan annotation approach and untargeted workflow

Annotation of the UNIGE-FIA-MS and UNIGE-LC-MS datasets was performed using MzAdan with default parameters: an absolute intensity threshold of 500 cps and 1% relative intensity, an intensity range of 1 to 100% for the deisotoping filter, and the monoisotopic validation enabled. The default annotation set, consisting of three adducts (ammonium, sodium, and potassium), two neutral losses (ammonia and water), and dimers and trimers, was used with an absolute tolerance of 10 mmu. MzAdan also considers combinations of mass differences resulting in a much higher number of possible forms. Lastly, only positive mode data were considered.

#### Evaluation of XCMS–CAMERA feature detection and grouping performance

Chromatographic feature detection for the 52 standard compounds mixture was achieved using the XCMS R package (v3.8.1) with the optimized parameters for UPLC/TripleTOF systems (method = centWave, ppm = 15, mzdiff = 0.01, peakwidth = c(5, 20), prefilter = c(3, 100), and snthresh = 6). CAMERA was then used for chromatographic deconvolution through retention time–based feature grouping using the groupFWHM function with default parameters.

Automated optimization of XCMS parameters was performed with the Isotopologue Parameter Optimization tool (v.1.14.0). Optimization was performed twice: without (IPO 1) and with (IPO 2) consideration of the XCMS prefilters option (ESM Table S5).

#### Comparative analysis of XCMS-based annotation software

The performances of several XCMS-based annotation tools, including CAMERA (v 1.42.0), findMAIN (v.1.2), and CliqueMS (v.1.0.1), were benchmarked on the both the 52 and the 35 metabolites contained in the standard mixture and the urine sample, respectively. The XCMS R package (v3.8.1) was used for peak finding with its parameters set to the recommended values for HPLC–HRMS systems. The influence of low-abundance features on analyte detection and annotation quality was assessed by varying the S/N from 1 to 24. Furthermore, the “prefilters” option was either enabled and set to its default values or entirely disabled. Lastly, a set of 12 common annotations ([M+Na+K-H]^+^ was excluded) was created for each software tool to allow for their direct comparison. The parameters and annotation sets used are detailed in Tables S6 to S13 (see ESM). Additionally, since mzAdan was not designed to process XCMS output, a Python script was written to automatically export the pseudo-spectra to mgf files.

## Results and discussion

### Ion processes and general considerations

In ESI, analyte ions are often formed by the addition or removal of protons to generate [M+H]^+^ ions in positive mode and [M−H]^−^ ions in negative mode, but a variety of other common ion source processes are known, including the (1) addition of different charged species, for example NH_4_^+^, Na^+^, and K^+^ in positive mode and CO_2_H^−^ and CH_3_CO_2_H^−^ in negative mode; (2) loss of small stable neutral molecules such as H_2_O, CO_2_, and NH_3_; and (3) formation of multimers such as [2M+H]^+^, [2M+Na]^+^, and [3M+H]^+^. However, reactions of ions with co-eluting analytes and background ions (solvents, contaminants) have also been reported, and these processes can occur individually or in combination, generating more complex species.

The general approach to annotation is to examine pairs of ions and determine if the mass difference corresponds to a known adduct or fragment. For example, a mass delta of 21.9819 corresponds to the difference between [M+H]^+^ and [M+Na]^+^, i.e., Na-H, and establishes a relationship between the ions. However, we have frequently noted the presence of singly charged ions apparently corresponding to the addition of several charged species which we ascribe to the replacement of labile protons by species such as Na and K, with subsequent addition of a single charged species, e.g., [M-H+Na]H^+^, [M-2H+2Na]H^+^, etc. The generic form of these ions is [M+*n*Na-(*n* − 1)H]^+^ with each member differing by 21.9819, and we note that the first member of this series (*n* = 1) cannot be distinguished from [M+Na]^+^. When *n* is greater than one, the adduct species can differ so, for example, [M+Na+K-H]^+^ is also possible with forms containing K differing by 37.9559. Although forms with Na and K are the most common, other species are also found. For example, as indicated below, we have observed ions including Ca, but since Ca has two charges, there is no need for the additional charged species, i.e., [M-H+Ca]^+^ is also possible and differs from [M+H]^+^ by Ca-2H which is 37.9464, a difference of 9.5 mmu from the potassium form. As multimers will often have more labile protons, the number of possible replacement forms will increase for larger multimers.

These observations emphasize the difficulty of generating a definite list of mass deltas since the actual forms will depend on the nature of the analyte (number of labile protons, functional group stability), its concentration, the available charged species (Na^+^, K^+^, Ca^2+^), background ions, and co-eluting species. To avoid using a large comprehensive list and increase the chances of random matches, we use a minimal list and examine the deltas from all detected ions, i.e., we trace paths through the spectrum.

Examining mass differences is most effective if the peaks are formed from the same analyte, and hence, there is a tendency to rely on chromatographic processing to extract spectra that are assumed to be pure. However, co-elution is inevitable so elution times and chromatographic profiles may be unreliable in complex samples or with noisy peaks. Thus, an efficient algorithm must handle mixed spectra. Although mass accuracy is very reliable and consistent within the same spectrum, the presence of analytes that are related or have similar masses may result in ions being incorrectly linked. Our approach is to look for [M+H]^+^ candidates within a spectrum based on the mass values and to validate the selection chromatographically if needed. We also retain low-intensity peaks since these may be critical in building the ion paths. These concepts are illustrated in the following sections.

### mzAdan annotation tool

As discussed above, protonated analyte ions provide informative elemental formulae and are essential for reliable compound identification via spectral library matching or de novo interpretation. In metabolomics, a variety of “open-source” annotation software is currently available for untargeted analysis of LC-MS data, but many are either packaged as all-in-one solutions (e.g., XCMS–CAMERA and MS-DIAL) or are explicitly designed to be used with specific chromatography processing tools or frameworks, such as XCMS and MzMine2. A new annotation tool, called mzAdan, was created to offer a more flexible, platform-agnostic, chromatography-independent solution that is capable of processing complex mixed mass spectra. MzAdan is a graph-based tool that is intended to process batches of high-resolution mass spectra, explore convoluted annotation networks, and identify protonated analytes using a ranking system based on explained total ion current (TIC) and peak mass relationships. The tool was also designed to be quickly incorporated in LC-MS untargeted workflows for the analysis of small molecular weight compounds, including metabolites.

The annotation algorithm initially considers every spectral peak as a putative [M+H]^+^ candidate and parses the mass spectrum in search of *m*/*z* shifts corresponding to known annotations. Unlike other approaches, mzAdan uses annotation sets containing a few frequently observed mass shifts but considers combinations which allows the formation of deep annotation networks. These sets are fully customizable and contain by default the mass deltas of three common LC-MS adducts (NH_3_, Na, K) as well as two neutral losses (–H_2_O, –NH_3_). Multiply charged ions are not considered since these are less likely for low molecular weight analytes and can lead to errors. The annotation process is repeated until the whole mass spectrum has been parsed and a graph is created where the nodes are peaks, and the edges connecting the nodes correspond to the annotations. Specifically, mzAdan uses weighted, directed graphs to store information, such as the quality (mass error) and the direction of the annotations. Generally, the graph contains groups of interconnected peaks (clusters) which are inspected by a ranking algorithm in order to identify the most likely [M+H]^+^ candidates based on the TIC explained and peak connectivity. In each cluster, the ranking algorithm considers each peak and sums the intensity of all peaks related through one or more annotations. Finally, the best ranking candidates, those with the highest explained TIC, are recorded for every cluster generated. If multiple candidates are proposed for a single cluster, which can happen if their explained TIC fraction is identical, their connectivity can be used to discard the less likely protonated form. In addition, a set of three informative indexes is generated for each candidate: the cluster global connectivity (CGC), cluster intensity coverage (CIC), and cluster count coverage (CCC). CGC corresponds to the number of ions contained in the cluster, while CIC and CCC represent the percentage of the spectrum TIC and peak count explained by these ions. The higher the index values, the more likely a candidate is to be corresponding to a real analyte.

### Illustration and evaluation of mzAdan annotation and clustering strategy

**Fig. 1:**
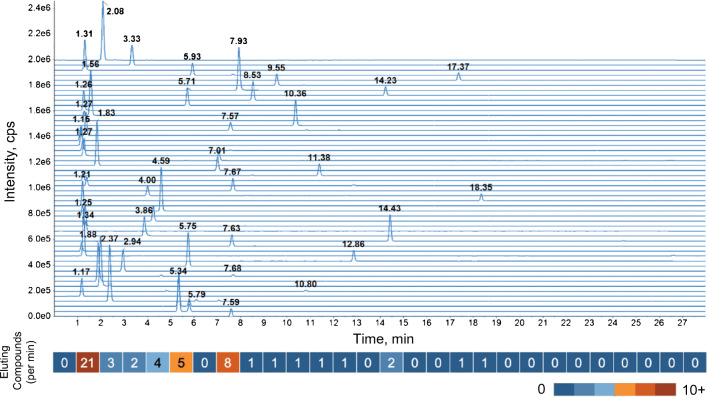
Extracted ion profiles for the 52 standard compounds in the UNIGE-LC-MS dataset (see ESM Table S2 for details). The number of eluting compounds per minute is displayed below the chromatograms

For the UNIGE-LC-MS data (Fig. 1), many peaks elute close to the void volume with minimal separation and several are chemically related, e.g., amino acids, creatinine, and creatine (ESM Table S2). The region between 1.3 and 1.4 min contains 5 analytes and is used to illustrate the annotation challenges outlined above and the mzAdan approach.

**Fig. 2:**
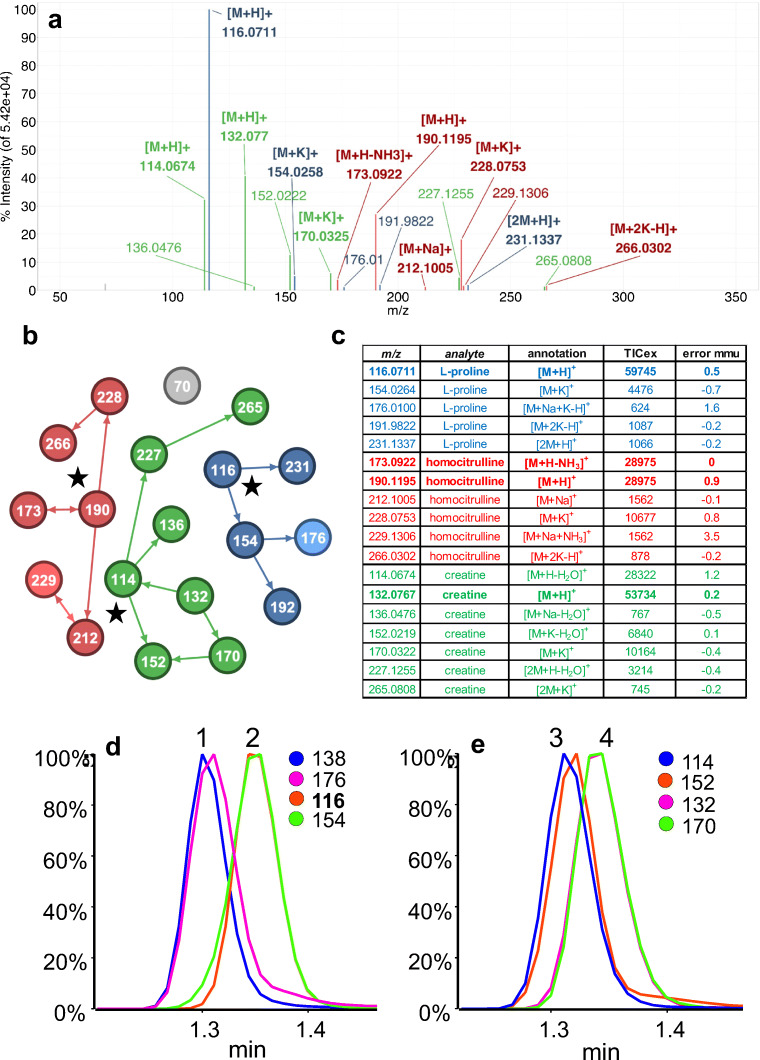
**a** Annotated, background-subtracted, deisotoped, and thresholded full scan spectrum of the UNIGE-LC-MS peak at 1.35 min. **b** Graph generated by mzAdan. The nodes correspond to related masses (nominal values are used for legibility) and arrows indicate the relationship direction and the most likely [M+H]^+^ candidate is marked with a star (see text for details). **c** Major ion assignments, explained intensity (TICex), and mass errors for the network clusters. Errors are relative to the elemental formula derived from the annotation. **d** Extracted ion chromatograms of [M+H]^+^ and [M+K]^+^ for 1: trigonelline (*m*/*z* 138.0550, 176.0108, RT 1.30 min) and 2: l-proline (*m*/*z* 116.0706, 154.0265, RT 1.35 min). **e** Extracted ion chromatograms of [M+H]^+^ and [M+K]^+^ for 3: creatinine (*m*/*z* 114.0662, 152.0221, RT 1.31 min) and 4: creatine (*m*/*z* 132.0768, 170.0326, RT 1.34 min)

Figure 2a shows the UNIGE-LC-MS background–subtracted spectrum of l-proline from a full width half maximum window centered at RT = 1.35 min after deisotoping and thresholding. Because of chromatogram crowding, even this simple processing results in a spectrum containing nineteen *m*/*z* values from at least three compounds which have been annotated and colored.

Figure 2b shows the graph generated by mzAdan using a 10-mmu tolerance window and the default annotation set of three adducts (NH_3_, Na, K) and two neutral losses (H_2_O and NH_3_), as well as dimers and trimers. The nodes correspond to *m*/*z* values (nominal values used for legibility) that are linked by arrows indicating parent–child relationships such as [M+H]^+^ → [M+Na]^+^. The singleton ion is ignored since there is no additional evidence of its identity, but it is likely a [M+H]^+^ from a background compound or low concentration analytes. For clusters of two ions, annotation usually identifies the [M+H]^+^, except for a delta mass of 17.0274 which can be [M+H]^+^ → [M+NH_4_]^+^ or [M+H]^+^ → [M+H-NH_3_]^+^ (an adduct or a loss) and is indicated by a bidirectional arrow (e.g., 173/190 and 212/229). For clusters of three or more ions, the algorithm examines each ion and calculates the intensity sum and establishes the connectivity of the ions it can explain. For example, 116 in the blue cluster can explain 154 and 231 (connectivity = 2), but 231 can only explain itself (connectivity = 1). The ion with the largest explained TIC, and connectivity in the case of a tie, is used as the [M+H]^+^ of the underlying analyte.

Figure 2c tabulates the mass values, assignments, explained TIC, and mass errors for ions in the three major clusters. For homocitrulline, the ions at *m*/*z* 173.0922 and 190.1195 explain the same TIC, but the latter has a higher connectivity and is selected as the [M+H]^+^ candidate. All assignments are reasonable and the mass errors consistent (mean = 0.33, median = − 0.05, stdev = 1.0) except for 176.0100 (error = 1.6 mmu) and 229.1306 (error = 3.5 mmu). As shown in Fig. 2d, peak 1, the 176 elution profile matches that of 138 and corresponds to the [M+K]^+^ of trigonelline (C_7_H_7_NO_2_, [M+H]^+^ 138.0550, [M+K]^+^ *m*/*z* 176.0108) and is within the mass tolerance window of the [M+K+Na-H]^+^ of proline (C_5_H_9_NO_2_, [M+K+Na-H]^+^ *m*/*z* 176.0084). The assignment for *m*/*z* 229.1306, [M+Na+NH_3_]^+^, is within the error tolerance, but there is no evidence of the corresponding [M+NH_4_]^+^ ion. The extracted ion chromatogram shows that it occurs when both creatinine (C_4_H_7_N_3_O, MW 113.059) and proline (C_5_H_9_NO_2_, MW 115.063) co-elute, suggesting the possibility of a heterodimer with the composition C_9_H_16_N_4_O_3_, MW 228.1222 and [M+H]^+^ 229.1295, an error of 1 mmu. Further investigation in this region reveals the presence of a second heterodimer between creatine and homocitrulline (C_11_H_24_N_6_O_5_, MW 320.1808) from a weak [M+H]^+^ ion at 321.1881.

A similar problem is illustrated in Fig. 2e. The software identifies *m*/*z* 132.0767 as an [M+H]^+^, which corresponds to creatine, based on a [M+K]^+^ adduct and water losses from the [M+H]^+^ and [M+K]^+^ ions. However, the XICs show two compounds: the second, represented by *m*/*z* 114.0679 and *m*/*z* 152.0219, is creatinine which differs from creatine by the elements of water, H_2_O. Since the elemental composition of creatinine and the water loss from creatine are identical, correct interpretation can only be solved by chromatography.

These examples show that mass measurements are reliable and consistent and can be used to flag potential errors, but chromatography can be essential to clarify ambiguous cases and low-intensity peaks may be important for correct interpretation. Furthermore, species such as the heterodimers will not have profiles that match any of the related ions. Hence, in approaches that rely on chromatographic peak picking, careful parameter choice is key and the chromatography and mass accuracy must match.

Overall, the total number of [M+H]^+^ candidates was reduced from 19 to 4 and the clusters containing the protonated forms of homocitrulline, l-proline, and creatine explained, respectively, 20%, 42%, and 37% of the mass spectrum TIC (CIC), and 6 (32%), 5 (26%), and 7 (37%) of the total peak counts (CCC) (ESM Table S14).

**Fig. 3:**
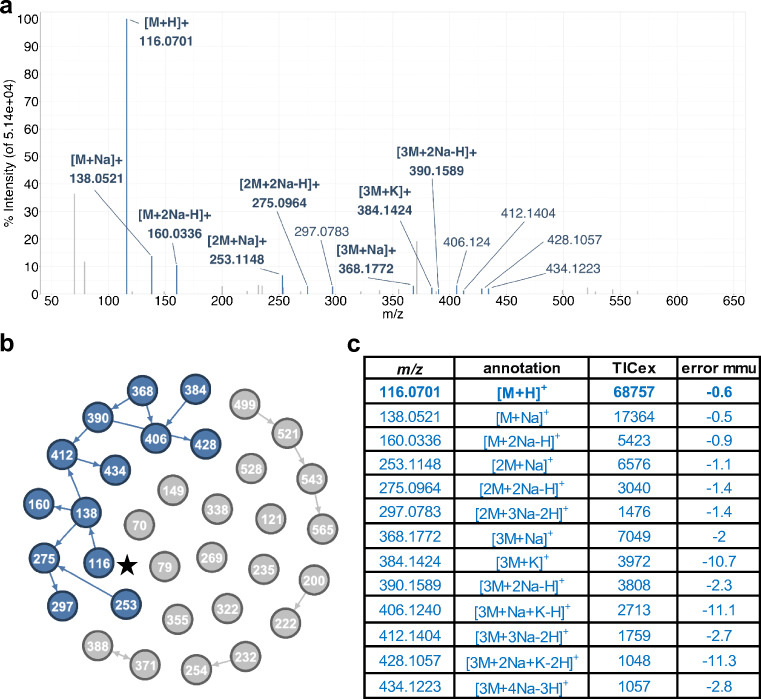
**a** Annotated, background-subtracted, deisotoped, and thresholded full scan spectrum of l-proline from the UNIGE-FIA data. **b** Network graph generated by mzAdan. Nodes correspond to related masses (nominal values used for legibility) and the arrows indicate relationship direction (see text for details). **c** Initial ion assignments, explained intensity (TICex), and mass errors for the main cluster. Errors are relative to the elemental formula derived from the annotation

For comparison, the positive mode spectrum of l-proline obtained by FIA with mzAdan annotation using the default parameters and an adduct mass difference tolerance of 10 mmu is presented in Fig. 3. Under these conditions, the spectrum showed extensive adduct formation and the annotation generated six clusters from the 33 filtered peaks: one with 13 peaks, one with 4, three with 2, and 10 singletons. The largest cluster contained the protonated form of l-proline, explained 61% of the mass spectrum total ion current and 13 (39%) of the total peak count, and showed dimers, trimers, and multiple replacement forms including combinations of Na and K (see ESM Table S15). However (Fig. 3c), the mass accuracy shows a wider spread (mean = − 3.75, median = − 2.0, stdev = 4.22) mainly due to the ions apparently corresponding to trimers with K adducts. This, combined with the observation that there are no other annotations with potassium, requires an alternate explanation, in this case adduction with Ca rather than K (adduct mass difference 9.5 mmu) as described previously, giving [3M+Ca-H]^+^, [3M+Na+Ca-2H]^+^, and [3M+2Na+Ca-3H]^+^ (see ESM Table S15 for exact masses and errors), and the mass accuracy statistics was as follows: mean − 1.7 mmu, median − 1.8, stdev = 0.78. With this information, the cluster of Na adducts starting at *m*/*z* 499.2041 can be identified as the equivalent tetramer ions: [4M+Ca-H]^+^, [4M+Na+Ca-2H]^+^, [4M+2Na+Ca-3H]^+^, and [4M+3Na+Ca-4H]^+^ with errors around − 3 mmu (ESM Table S15). This again illustrates the reliability of mass accuracy and underscores the need for flexible annotation sets and a tool that can be used interactively.

Comprehensive analysis of the 408 and 52 analytes of the UNIGE-FIA-MS and UNIGE-LC-MS datasets showed that the number of spectral features, and thus the number of [M+H]^+^ candidates, could be reduced by about 22% and 31%, respectively, after deisotoping and by an additional 41% and 51% following annotation with mzAdan. Clusters containing the protonated form explained a significant percentage of the TIC with averages of 69% and 67% for the UNIGE-FIA-MS and UNIGE-LC-MS, as well as 39% and 53% of the total number of peaks per spectrum. Despite the rather small size of the annotation sets and the presence of numerous ions derived from co-eluting compounds in LC, significant fractions of all mass spectra were explained in both datasets. Furthermore, although FIA is not commonly used in metabolic studies, the higher complexity of the data did not affect the performance of mzAdan.

MzAdan’s ranking system correctly identified the majority of protonated analytes in their clusters but failed to identify the correct [M+H]^+^ for six of the 408 standard analytes of the UNIGE-FIA-MS dataset (ESM Table S1) and three early eluting analytes (carnosine, trigonelline, and creatinine) of the 52 compounds of the UNIGE-LC-MS dataset. Incorrect identification for the FIA dataset was mainly caused by the formation of clusters containing highly interconnected ion networks including nonanalyte-related species or unexpected adduct combinations. In the LC data, overlapping elution profiles of analytes with forms close in mass was the only cause of failure. As shown above, the masses of creatinine (MW = 113.0589 Da, RT = 1.31 min) and creatine (MW = 131.0695 Da, RT = 1.30 min) differ by 18.0106 amu, which led to creatinine being annotated as the [M+H-H_2_O]^+^ of creatine (Fig. 2). Similarly, trigonelline (MW = 137.0477 Da, RT = 1.30 min), which eluted close to l-proline (MW = 115.0633 Da, RT = 1.35 min), was annotated as the [M+Na]^+^ of l-proline (*m*/*z* 138). Moreover, the clusters of two co-eluting compounds may be merged into one that contains both protonated forms when their adducts match the same peak in the mass spectrum. For instance, the [M+H-NH_3_-H_2_O]^+^ of carnosine (*m*/*z* 192.0743, RT = 1.16 min) and the [M+Na]^+^ of 1-methylhistidine (*m*/*z* 192.0743, RT = 1.17 min) lead to merging the two corresponding clusters and identification of 1-methylhistidine as the single most probable [M+H]^+^ candidate.

As shown above, mzAdan relies on stable mass accuracy, which is also a good measure of result confidence and can flag the presence of difficult cases such as unexpected charge agents and heterodimer formation. Nonetheless, careful LC-MS peak selection ensures that related peaks are correctly grouped and small important peaks retained. In contrast, the popular XCMS tool is based on chromatographic feature detection (RT, *m*/*z*) with subsequent adduct annotation software generating pseudo-spectra for RT windows. This approach has several drawbacks: (i) chromatographic peak width and symmetry are not constant over the LC-MS run; (ii) LC peaks are best detected using at least 10 data points; (iii) in complex samples, analyte co-elution is common; and (iv) a low-intensity peak may be missed or misassigned due to noisy traces. Since mzAdan can be incorporated into a XCMS-based workflow, we evaluated the peak-picking parameters and compared our results with other annotation tools.

### Evaluation of XCMS–CAMERA peak picking and feature grouping with the LC-MS dataset

Conventional LC-MS–based workflows use peak picking, alignment, and grouping, before annotation. Peak-picking tools generate lists of features (unique *m*/*z*–retention time pairs) that correspond to single chromatographic peaks or sets of peaks aligned across multiple samples. Features are grouped into pseudo-spectra based on retention time and chromatographic peak shape similarities. Optimization of the numerous parameters for peak picking and grouping is critical since these can drastically affect the number and quality of the features detected and their correct grouping in the pseudo-spectra. In XCMS, several parameter settings can be adjusted, including peak width, *m*/*z* tolerance, and prefilters, as well as ion intensity and signal-to-noise ratio thresholds. Selection of the optimum settings for a distinct LC-MS run is customarily achieved via manual examination of the LC-MS performance following extensive parameter testing which can be challenging and time-consuming, especially for inexperienced users. Thus, it is good practice to start with the recommended parameters optimized for specific chromatographic systems and mass spectrometers provided by the XCMS online platform [20, 21].

The XICs of the 52 protonated forms in the raw data presented in Fig. 1 can be divided into three distinct regions: region (1) from 1 to 2 min showed strong co-elution of 21 compounds, region (2) from 2 to 8 min showed moderate co-elution of 22 compounds, and region (3) ranging from 8 to 19 min showed low co-elution with nine compounds in 11 min.

With the recommended XCMS parameters optimized for HPLC–HRMS platforms, the 52 mix generated a total of 665 features (1–18.5 min), which were collected in 228 pseudo-spectra by CAMERA using only the retention time–based peak grouping function (groupFWHM). Of these hundreds of features, 50 corresponded to the protonated forms of one of the 52 compounds. Eluting at 4.83 and 7.68 min, respectively, 3-chlorotyrosine and 5′-methylthioadenosine were not identified by XCMS. Of the 50 detected compounds, 44 appeared in individual spectra, while the remaining six were grouped with one or two additional metabolites. In total, five composite mass spectra were generated in a narrow time window of 11.4 s, starting at 1.16 min. Nonetheless, the previously discussed problematic features associated with co-eluting creatinine (M14) and creatine (M15), as well as trigonelline (M13) and l-proline (M16), were contained in different pseudo-spectra. Although retention time feature grouping may solve some co-elution issues, the process remains challenging particularly early in the chromatogram and with noisy signals.

We used a list of 13 common adducts (ESM Table S4) to manually annotate the 52 standard analyte mixture mass spectra from PeakView (ESM Table S16) and XCMS–CAMERA (ESM Table S17) where 119 and 33 ions were respectively annotated. XCMS–CAMERA did not generate a pseudo-spectrum for two analytes. Comparison of the results revealed a substantial loss of 86 (72%) compound-related features in the pseudo-spectra. The [M+NH_4_]^+^, [M+K]^+^, and [M+H-NH_3_]^+^ showed decreases of, respectively, 25%, 50%, and 50% in the number of annotations, and other adducts had even more significant losses. Of the 25 [M+Na]^+^ ions identified in the raw data, only two were part of the pseudo-mass spectra and none of the 19 [M+2K-H]^+^ and 9 [M+Na+K-H]^+^ annotated peaks were found in the pseudo-spectra. Although detection of fewer features resulting in mostly protonated analytes is viewed as beneficial, the absence of these additional signals, including adducts, in-source fragments, and oligomers, adversely affects annotation tools that rely on peak connectivity. In addition, if the relationship between protonated ions and other forms is not recognized, the latter may be considered as additional unique compounds.

The first region of the chromatogram presented in Fig. 1 contained 44 annotated ions, but only 20% of the corresponding features were found in XCMS pseudo-spectra. The second region contained 42 annotations of which XCMS detected 24%, while, in contrast, the third region showed a much lower loss and 58% of the 33 annotations were detected. Additionally, for the first, second, and third regions of the chromatogram, respectively, 67%, 55%, and 22% of the analytes had none of the 13 annotations in their pseudo-mass spectra.

As background-subtracted mass spectra may still contain ions from neighboring chromatographic regions, it is possible that some annotations were due to mixed mass spectra, but XICs of the annotated features revealed that 97% eluted with the corresponding analyte. Of the four misannotated peaks, three were identified as neutral losses ([M+H-NH_3_]^+^, [M+H_2_O]^+^) and one as sodium potassium adducts ([M+Na+K-H]^+^). Since only a small fraction (3%) of annotated ions did not co-elute with their corresponding analyte, the difference in retention time could not explain the considerable loss of the analyte-derived features discussed above. Hence, we suspected that one or more XCMS parameters were preventing the detection of low-abundant features.

### Optimization of XCMS parameters for feature detection

An R package named IPO was recently developed to automatically optimize a predefined set of XCMS parameters. The tool was benchmarked against the traditional manual tuning workflow [21] and was shown to produce more robust results, in terms of cross-sample feature detection. However, this study also cautioned about unrealistic values that might be suggested for complex biological samples when the tool is used in an unsupervised manner. In our hands, using this tool to optimize settings for the 52 standard compounds mixture was initially unsuccessful and the number of detected analytes was reduced to 38, and the number of annotated features decreased to 22.

Manually tuning the XCMS peak width and *m*/*z* tolerance parameters showed no significant increase in analyte-related feature detection (ESM Table S18) in fact increasing or decreasing the settings caused a rapid decrease in XCMS feature detection performance. In contrast, the signal-to-noise ratio and “prefilters” options had notable impact on the detection of analyte and analyte-related features (ESM Table S18). By default, prefilters are automatically applied to discard low-intensity chromatographic regions to decrease both processing time and the number of low-abundance features. To be considered a potential feature of interest by XCMS, an ion corresponding to a distinct mass trace must be present in *n* scans with an intensity higher than a threshold, *k*; otherwise, it is discarded by the prefilters. The default values of these parameters are a minimum of three scans and an intensity of 100 cps, i.e., *n* = 3, *k* = 100, or (3100). Disabling the prefilters increased the number of detected features from 665 to 1700 and raised the number of pseudo-spectra from 228 to 309. Eventually, all 52 protonated forms were detected, and the number of annotated features increased from 33 to 161, while the number of pseudo-spectra containing the 52 protonated forms and one or more annotated features increased from 24 to 46.

**Fig. 4:**
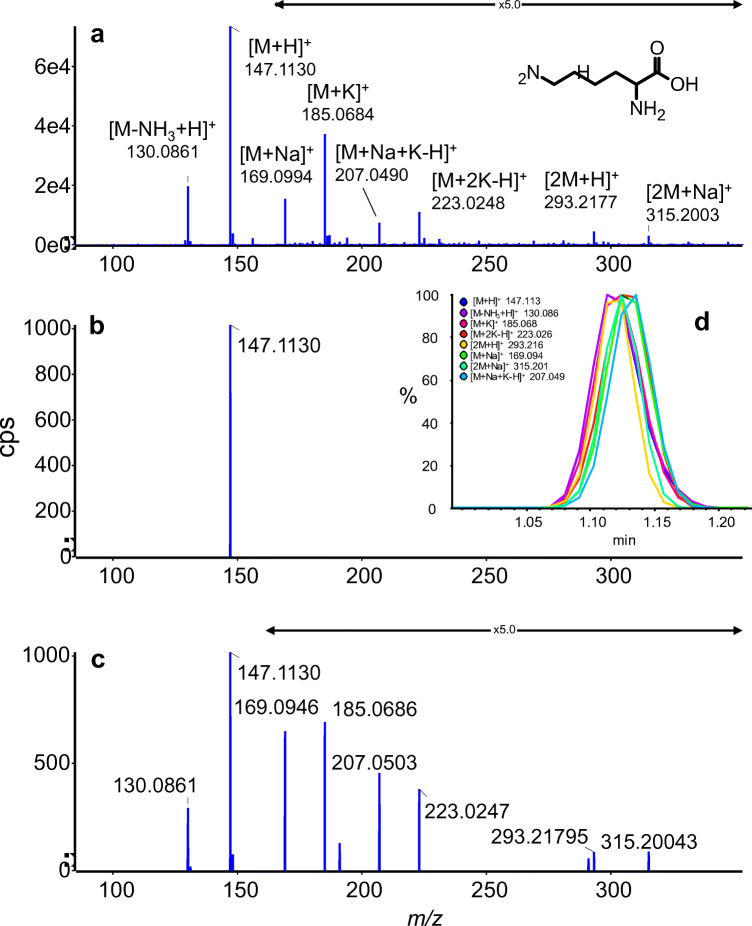
Manually annotated mass spectra of l-lysine (C_6_H_14_N_2_O_2_) considering a set of 12 annotations: [M+H-H_2_O]^+^, [M+H-NH_3_]^+^, [M+H]^+^, [M+NH_4_]^+^, [M+Na]^+^, [M+K]^+^, [M+2Na-H]^+^, [M-H+2K]^+^, [2M+H]^+^, [2M+K]^+^, [2M+Na]^+^, and [3M+H]^+^. Spectra were either extracted using PeakView (Sciex) (**a**) or generated with XCMS–CAMERA. Only monoisotopic peaks with intensities above 1% and 500 cps were annotated in PeakView, while XCMS pseudo-spectra were annotated “as is.” Only the protonated form of l-lysine was detected using XCMS with the default prefilter settings (**b**), but disabling the prefilters option resulted in more features, including many adducts (**c**). A total of eight peaks were annotated with both PeakView and XCMS with prefilters off. The extracted ion chromatograms of the annotated features show that they are slightly displaced (**d**)

Although IPO allows prefilter optimization, this option was not discussed in either the initial article or the subsequent performance assessment [22]. IPO was rerun to optimize the prefilters along with XCMS main settings (peak width, tolerance, *m*/*z* diff, and S/N) which caused most parameters to be closer to the recommended values, except for the S/N and prefilters which were set to their minimum values (ESM Table S5). These settings allowed detection of the 52 features corresponding to the standard analytes and 206 adducts and neutral losses. Only a few XCMS parameters, especially signal-to-noise ratio and the prefilter settings, significantly affected the number of detected analytes and compound-derived signals, and since the recommended parameters were comparable to that of IPO, all were used “as is,” except for prefilters and S/N. The impact of the prefilters on the detection of compound-derived features is illustrated with l-lysine in Fig. 4 and *N*-acetyl-l-phenylalanine in Fig. S1 (see ESM). These settings are examined further below to evaluate the consequence of discarding low-abundant features on XCMS-based annotation software.

### Comparative analysis of XCMS-based annotation tools for LC-MS analysis of the standards mixture and urine sample

MzAdan was incorporated into a conventional LC-MS untargeted analysis workflow with the widely used XCMS platform and evaluated using a mixture of 52 standard compounds and 35 selected metabolites present in a human urine sample. We compared the performance of mzAdan with that of CAMERA, findMAIN, and CliqueMS with different XMCS parameter values for S/N and the prefilters and considered the deficiencies of each software. Even though a wide range of annotation tools is currently available for LC-MS metabolomics analysis with XCMS, these three tools were selected for their ability to also process single samples.

**Fig. 5:**
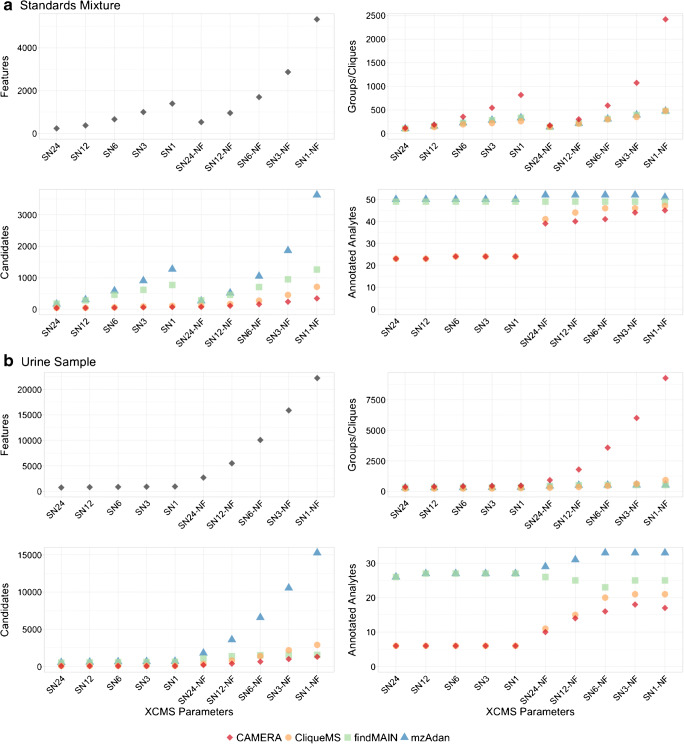
Summary of four XCMS-based annotation software packages for the standards mixture (**a**) and urine sample (**b**). These tools were tested using data generated by XCMS with different S/N thresholds (sn1–sn24), and the prefilters enabled and set to default values or entirely disabled (nf). The number of features detected by XCMS increased with lower signal-to-noise ratios and the prefilters disabled, and the number of pseudo-spectra generated, [M+H]^+^ candidates, and metabolite annotations increased. The number of pseudo-spectra generated with CAMERA using its peak shape-based feature grouping algorithm was consistently higher than CliqueMS, while CliqueMS and CAMERA performed worse than findMAIN and mzAdan for both samples and sets of parameters tested, though disabling the prefilters did improve performance. findMAIN and mzAdan consistently produced higher numbers of [M+H]^+^ candidates

The four software packages were tested with the set of annotations used previously, but without [M+Na+K-H]^+^, and ten lists of features generated by XCMS with different parameter values for the signal-to-noise ratio and prefilters option. The S/N was varied from 1 to 24 (sn1–sn24), and the prefilters were either enabled and set to default values (scans = 3, intensity = 100) or disabled (nf). These settings were specifically chosen to assess the influence of low-abundant features on correct analyte annotation. Moreover, as recommended for findMAIN and CliqueMS, respectively, the three and five best scoring annotations were considered. Detailed results for the mix of 52 standards and each set of parameters tested are illustrated in Fig. 5a.

To evaluate the reproducibility of each software, each analysis was repeated three times for each dataset, software, and set of parameters tested. All annotation tools returned identical outputs for the three replicates, except CliqueMS which showed limited reproducibility. Features corresponding to the protonated form of some analytes were inconsistently annotated in both the urine sample and the standard mixture depending on the parameter sets tested (ESM Fig. S2). The CliqueMS peak grouping algorithm was eventually identified as the cause of these discrepancies as several features were repeatedly grouped in different cliques which affected the downstream annotation process. Since no replicate showed significantly better performance, we arbitrarily selected the results of the first analyses for the following comparison.

Considering the detection of known analytes, mzAdan showed the best performance for the standard mixture closely followed by findMAIN. Both tools correctly annotated the majority, if not all, of the features corresponding to the 52 protonated forms. In contrast, CAMERA and CliqueMS showed overall lower performance mainly related to the set of parameters tested but especially with the prefilters set to default values. Two sets of features (SN6 and SN6-NF) are discussed in detail below to explore the advantages and shortcomings of each annotation tool.

The main factor limiting the performance of the annotation software is detection of specific adduct features associated with the analytes of interest. Out of the 52 metabolites in the standard sample, 50 protonated forms were detected in SN6 compared with SN6-NF where all 52 were identified (ESM Tables S19 to S23).

**Fig. 6:**
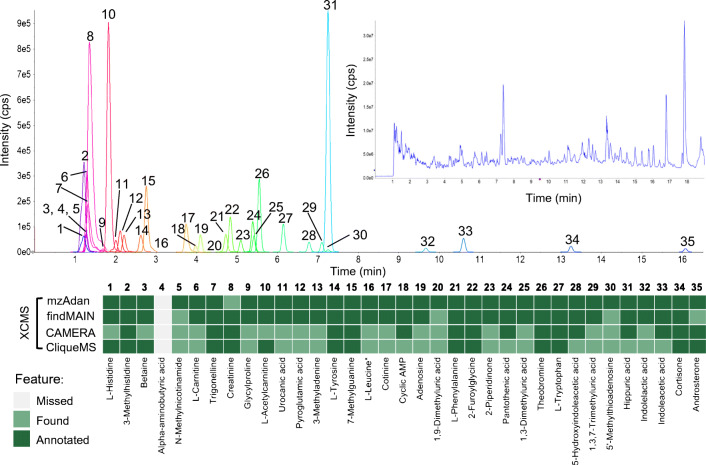
Results obtained for the annotation of 35 reference metabolites identified in the urine sample using mzAdan, findMAIN, CAMERA, and CliqueMS, with XCMS for feature detection and grouping. The entire chromatogram is shown (inset), as well as the extracted ion currents of each analyte and a table indicating analyte detection with the different software packages. No peaks were detected for alpha-aminobutyric acid and mzAdan assigned creatinine as a loss of water from creatine

The urine sample is far more complex with regard to the number of analytes, with many metabolites co-eluting and a large range of MS response. A representative chromatogram of the LC-MS analysis with extracted ion current profiles of the 35 selected metabolites is presented in Fig. 6. The four software packages were tested under the same conditions described for the standard mix and the results are presented in Fig. 5b and the outcome is summarized in Fig. 6. As before, MzAdan showed the best performance closely followed by findMAIN. Out of 35 metabolites, 27 protonated forms were detected in SN6, while in SN6-NF, 34 were identified (ESM Tables S24 to S28 for urine).

The annotation approaches used by CAMERA and CliqueMS share similarities that partly explain their lower performance compared with findMAIN and mzAdan. Both are highly dependent on peak connectivity and require two or more features to be linked by annotation for the molecular mass of the hypothetical neutral molecule to be computed. CAMERA computes putative neutral masses for each group of two or more adducts and then proposes the most likely one by summing adduct-specific frequency scores. In contrast, CliqueMS uses the log of adduct frequencies to propose the most likely molecular mass of a group of two or more adducts. The lack of a rational connection between the analytes’ protonated forms and co-eluting features prevented both software from computing these theoretical neutral masses for a significant number of analytes. In SN6, of the 50 and 27 metabolites detected by XCMS in the mixture of standards and urine samples, 26 and 21 could not be annotated by either package. In SN6-NF for the standard mixture, one analyte was misannotated and ten others received no annotation using CAMERA, while eight were not annotated with CliqueMS. Ultimately, both approaches appear to be strongly impacted by the composition of the pseudo-mass spectra as well as by the variety of adducts chosen for the annotation.

Examination of the mass spectra used for benchmarking the findMAIN and mzAdan approaches showed that several adducts detected by XCMS were absent from the CliqueMS and CAMERA pseudo-spectra containing the corresponding protonated analytes. In fact, CAMERA provides two feature grouping algorithms: one based on retention time (groupFWHM) and the other on peak shape correlation (groupCor). The groupFWHM function generated the pseudo-spectra used with findMAIN and mzAdan, and both functions were applied successively to produce the ones for CAMERA. CliqueMS uses its own feature grouping algorithm based on a similarity network to generate its pseudo-spectra. Both CAMERA and CliqueMS relocated several features to different pseudo-mass spectra and by doing so sometimes prevented the annotation of adducts which were required for computation of the analyte neutral mass. However, this issue was more prevalent for CAMERA.

Mahieu et al. [21] recently showed that adducts can produce EIC significantly different from the typical and expected Gaussian-like profiles and from the related species (see also Fig. 4d). Consequently, feature grouping based on peak shape correlation can lead to the segregation of many related signals. Figure S3 (see ESM) shows that decreasing the cutoff of the groupCor function can significantly reduce the number of pseudo-spectra and increase the number of annotated analytes.

FindMAIN considers every feature as one or several possible common ionization products (e.g., [M+H]^+^, [M+Na]^+^, [M+K]^+^) and uses a weighted scoring system based on explained intensity, mass accuracy, and isotope charge agreement to rank the most likely annotation (i.e., hypothetical molecular mass). In contrast to CliqueMS and CAMERA, this solution provides scored annotations to each peak in the mass spectrum, even in the absence of a relationship, other than with isotopologue ions. As a result, findMAIN performs well even when known adducts are absent from the spectrum and the systematic annotation of every spectral feature greatly increases the number of putative analyte candidates. It is recommended that only the top three candidates be considered, but then, several real molecular masses were ranked much lower. Consequently, keeping only the top scoring candidates can cause the loss of several correct annotations and considering lower-ranked ones may lead to an overestimation of putative metabolites in the samples.

MzAdan takes a similar approach to findMAIN, as it considers every feature as a possible protonated ion ([M+H]^+^). Although its annotation approach is designed to reduce the number of putative candidates through annotation and clustering of analyte-derived signals, the number of candidates remains higher than for the other annotation tools. Indeed, mzAdan does not compute likelihood scores for each annotation to discard the least plausible ones. Instead, the tool provides the whole list of candidates and several informative indexes (CGC, CIC, and CCC) that can be used to significantly reduce the number of putative analytes while keeping those that most likely correspond to metabolites. The higher the value of these indexes, the more likely an annotation will correspond to a real analyte.

## Conclusions

For many analytes, electrospray ionization generates protonated and deprotonated ions, but many different species are also observed including water or ammonia loss as well as ammonium, sodium, and potassium adducts. The presence of adducts is often related to trace amounts of these cations in the mobile phases and their intensities depend on the analyte and analysis conditions. For example, l-proline shows the presence of sodium and calcium adducts and multimers in flow injection analysis (Fig. 3), while mostly potassium adducts are observed with liquid chromatography (Fig. 2). A similar behavior is observed for most of the analytes investigated in the present study. In qualitative work for analyte identification (e.g., elemental formulae), adduct annotation has become an essential step in data processing. Considering combinations of water and ammonia losses, sodium, potassium, ammonia adducts, and multimers, about 50% or the TIC of the spectra can be annotated, but half of the signals remain unexplained. Many different software solutions have been described for metabolomics workflows but are integrated into rigid untargeted analysis pipelines or depend on the outputs of specific chromatographic peak-picking tools. MzAdan, a nonchromatography-based multipurpose desktop application, was developed for the annotation and exploration of convoluted high-resolution ESI-MS spectra. The tool annotates single or multiple spectra (in mgf format) using accurate mass with a customizable adduct annotation list and produces a list of [M+H]^+^ candidates. With recent instruments, accurate mass measurements are consistent and essential to differentiate the correct adduct (e.g., calcium versus potassium), but chromatography (extracted ion current profiles) can be helpful to clarify ambiguous cases. Low-intensity peaks are important for correct interpretation and can be missed by approaches that rely on chromatographic peak picking. The performance of MzAdan was evaluated with FIA-MS spectra of standards, and 402 of the 408 analytes could be correctly annotated as [M+H]^+^. MzAdan was incorporated in an untargeted LC-MS workflow with XCMS, applied to a 52 metabolite mix and human urine sample and benchmarked against three other annotation tools, CAMERA, findMAIN, and CliqueMS. XCMS with recommended parameter settings missed many important low-intensity features but, despite this, FindMAIN and mzAdan consistently produced higher numbers of [M+H]^+^ candidates than CliqueMS and CAMERA. With lower signal-to-noise ratios and disabled prefilters, the number of features detected by XCMS increased and the number of generated pseudo-spectra, [M+H]^+^ candidates, and metabolite annotations also increased. Analyte co-elution and composite spectra are a major challenge for adduct annotation software, and mzAdan was designed to handle mixed multi-analyte spectra.

While protonated molecules generate good quality MS/MS spectra by collision-induced dissociation, information is often limited with adduct precursor ions. This is important for data-dependent acquisition (DDA) workflows where precursors selected based on intensity may generate poor product ion spectra if adduct ions are chosen. However, this may not be true for other dissociation techniques, e.g., electron-induced fragmentation [23]. The ideal option would be the application of a real-time annotation filter capable of handling mixed spectra, such as mzAdan, with selection of the best precursor ions for a given fragmentation technique. In the selected reaction monitoring mode, largely used for targeted quantitative analysis, the analyst does not usually see changes in adducts of the analyte of interest, and this may cause limited assay performance and challenges in assay transfer. Finally, undetected adducts and complex forms such as heterodimers may change with conditions or system impurities and can confound applications such as metabolomics and lipidomics that rely on measuring changes across many samples.

Overall, electrospray ionization remains a complex process and the fact that a large part of the ESI spectra collected can still not be explained requires further investigations.

## Electronic supplementary material

ESM 1 (PDF 754 kb).

ESM 2 (XLSX 31 kb).

ESM 3 (XLSX 40 kb).

## Data Availability

The LC-MS raw data underlying this article are available in the Yareta research data repository, at 10.26037/yareta:wppmltdgkrh7bak2skce5alxzi.
