# Supplementary material for: Adduct annotation in liquid chromatography/high-resolution mass spectrometry to enhance compound identification
Source: Anal Bioanal Chem. 2020 Oct 29;413(2):503–17. doi: 10.1007/s00216-020-03019-3 (PMC7806579; doi:10.1007/s00216-020-03019-3)
Supplement: Supplementary file 1 — (PDF 754 kb). [file 216_2020_3019_MOESM1_ESM.pdf]

Analytical and Bioanalytical Chemistry

Electronic Supplementary Material

Adduct annotation in liquid chromatography/high-resolution mass spectrometry to enhance compound identification

Thomas Stricker, Ron Bonner, Frédérique Lisaek, Gérard Hopfgartner

Additional files available under 10.1007/s00216-020-03019-3.

Adduct annotation in liquid chromatography/high-resolution mass spectrometry to enhance compound identification

Thomas Stricker[1,3], Ron Bonner[2], Frédérique Lisaçek[3] and Gérard Hopfgartner[1*]

1. Life Sciences Mass Spectrometry, Department of Inorganic and Analytical Chemistry, University of Geneva, 24 Quai Ernest Ansermet, CH-1211 Geneva 4, Switzerland
2. Ron Bonner Consulting, Newmarket, ON, L3Y 3C7, Canada
3. Proteome Informatics Group (PIG), Swiss Institute of Bioinformatics and University of Geneva, 7, route de Drize, CH-1211 Geneva 4, Switzerland

* corresponding author

Supplementary Info

Table of Contents

Supplementary Figures 4

Figure S1: Manually annotated mass spectra of N-acetyl-phenylalanine, considering a set of 12 annotations. 4

Figure S2: Summary of the analysis of three replicates generated for both the standards mixture and urine sample with CliqueMS. The three replicates were acquired using XCMS with various signal-to-noise thresholds (sn1-sn24), and the prefilters option was either enabled and set to its default values or entirely disabled (nf). Certain features were not consistently grouped in the same clique, when a specific dataset was analysed several times with identical parameters. As the CliqueMS scoring system is strongly dependent on connectivity, differences in the content of the pseudo-spectra can affect the annotation of the metabolites. In the end, several analytes were annotated in only one or two replicates. 5

Figure S3: Summary of the analysis of CAMERA's features annotation and grouping performance for standards mixture (52 analytes) and the urine sample (35 target analytes). 6

Supplementary Tables: 7

Table S1: Summary of the 408 analytes producing [M+H]+ ions by flow injection analysis (UNIGE-FIA/MS). Analytes in yellow are the 6 analytes where mzAdan to failed to identify the $[M+H]^+$. 7

Table S2: Summary of the 52 metabolites of the LC-mixture (UNIGE-LC/MS). 17

Table S3: Summary of the 35 metabolites considered in the human urine sample. 19

Table S4: Annotation set used for the manual annotation of the standards mixture mass spectra, including PeakView data and XCMS pseudo-spectra. Mass differences are relative to the Molecular Weight. 20

Table S5: Default and optimised XCMS parameter settings obtained with IPO. 20

Table S6: Detailed parameters used for XCMS and CAMERA for the comparative analysis of XCMS-based annotation tools. 21

Table S7: Detailed parameters used for XCMS and CliqueMS for the comparative analysis of XCMS-based annotation tools. 21

Table S8: Detailed parameters used for XCMS, CAMERA and findMAIN for the comparative analysis of XCMS-based annotation tools. 21

Table S9: Detailed parameters used for XCMS, CAMERA and mzAdan for the comparative analysis of XCMS-based annotation tools. 23

Table S10: Annotation set used with CAMERA for the comparative analysis of XCMS-based annotation tools. 23

Table S11: Annotation set used with CliqueMS for the comparative analysis of XCMS-based annotation tools. 24

Table S12: Annotation set used with FindMAIN for the comparative analysis of XCMS-based annotation tools. 24

Table S13: Annotation set used with mzAdan for the comparative analysis of XCMS-based annotation tools. 24

Table S14: Candidate lists proposed by mzAdan for the data in Figures 2 and 3 25

Table S15: Peak annotation summary for L-proline by FIA-MS analysis (Figure 3) 26

Table S16: Relative intensities of the 13 adducts (including protonated forms) of the LC/MS analysis of the 52 analyte mix. Mass spectra were generated with PeakView (average at FWHM and background subtracted). Only annotations with correct retention times were considered (mzAdan and XIC). 27

Table S17: Relative intensities of the 13 adducts (including protonated forms) following manual annotation of the standards mixture mass spectra with features detected using XCMS with the recommended parameters, and pseudo-mass spectra were generated with CAMERA. 29

Table S18: Number of features corresponding to known analytes or adducts detected in the standards mixture with XCMS. 31

Table S19: Peak picking results for the standard mixture using XCMS with various signal-to-noise thresholds (sn1-sn24), and prefilters either enabled and set to their default values or entirely disabled (nf). 32

Table S20: Summary of the annotation of the standards mixture using XCMS-CAMERA with various signal-to-noise thresholds (sn1-sn24), and prefilters either enabled and set to their default values or entirely disabled (nf). 34

Table S21: Summary of the annotation of the standards mixture using XCMS-CliqueMS with various signal-to-noise thresholds (sn1-sn24), and prefilters either enabled and set to their default values or entirely disabled (nf). .. 36

Table S22: Summary of the annotation of the standards mixture using XCMS-FindMain with various signal-to-noise thresholds (sn1-sn24), and prefilters either enabled and set to their default values or entirely disabled (nf). .. 38

Table S23: Summary of the annotation of the standards mixture using XCMS-mzAdan with various signal-to-noise thresholds (sn1-sn24), and prefilters either enabled and set to their default values or entirely disabled (nf)............ 40

Table S24: Summary of the peak picking process of the urine sample using XCMS with various signal-to-noise thresholds (sn1-sn24), and prefilters either enabled and set to their default values or entirely disabled (nf)............ 42

Table S25: Summary of the annotation of the urine sample using XCMS-CAMERA with various signal-to-noise thresholds (sn1-sn24), and prefilters either enabled and set to their default values or entirely disabled (nf)............ 44

Table S26: Summary of the annotation of the urine sample using XCMS-CliqueMS with various signal-to-noise thresholds (sn1-sn24), and prefilters either enabled and set to their default values or entirely disabled (nf)............ 46

Table S27: Summary of the annotation of the urine sample using XCMS-FindMain with various signal-to-noise thresholds (sn1-sn24), and prefilters either enabled and set to their default values or entirely disabled (nf)............ 47

Table S28: Summary of the annotation of the urine sample using XCMS-mzAdan with various signal-to-noise thresholds (sn1-sn24), and prefilters either enabled and set to their default values or entirely disabled (nf)............ 48

Supplementary Figures:

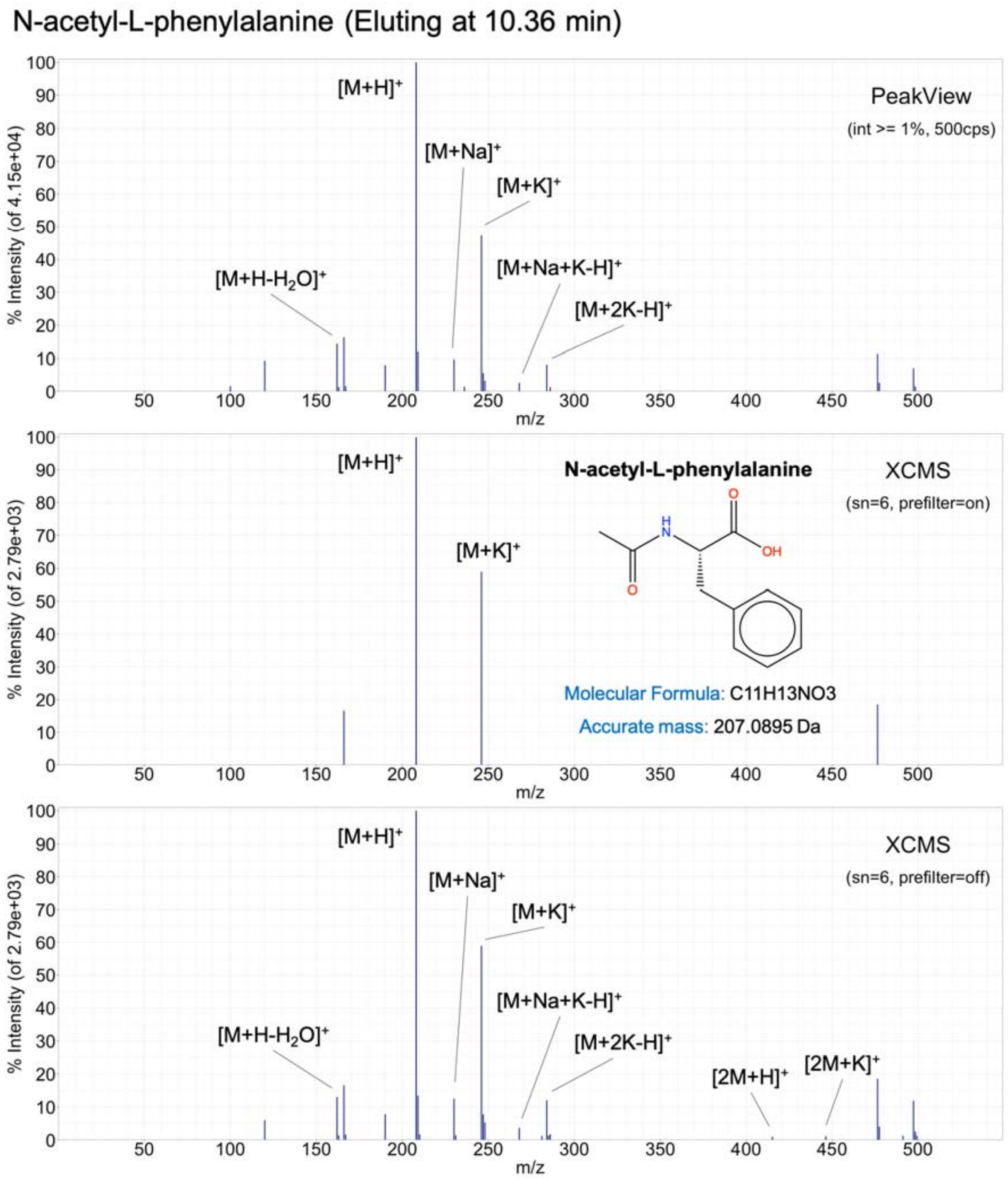

Figure S1: Manually annotated mass spectra of N-acetyl-phenylalanine, considering a set of 12 annotations. Mass spectra weregenerated using either PeakView (Sciex) or XCMS-CAMERA. Only monoisotopic peaks with intensities above 1% and 500 cps were considered for annotation in PeakView mass spectra, while XCMS pseudo-spectra were annotated as is. Disabling the prefilters option in XCMS allowed detection of a higher number of features, including many corresponding to adducts. A total of six and eight peaks were annotated with respectively PeakView, and XCMS with prefilters turned off. In contrast, only the protonated form and the potassium adduct of the N-acetyl-L-phenylalanine were detected using XCMS with prefilters enabled and set to their default values.

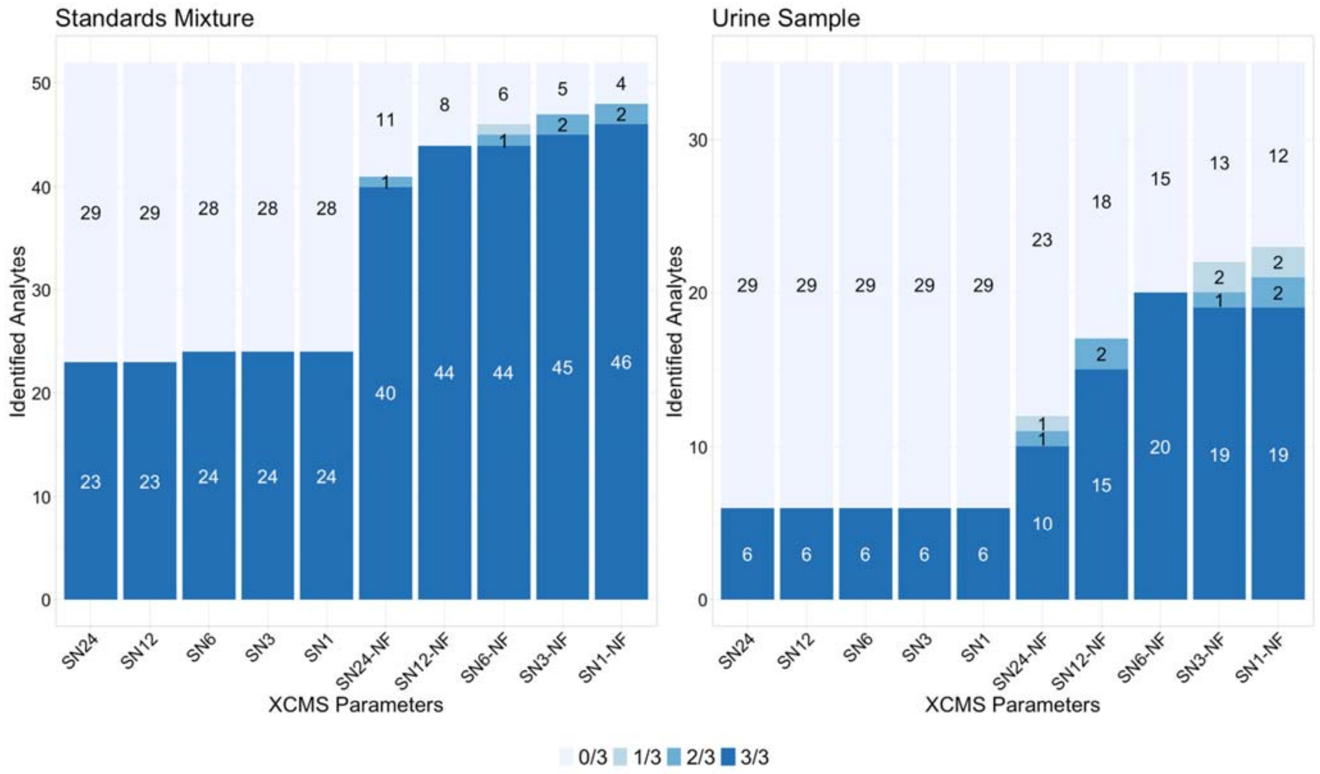

Figure S2: *Summary of the analysis of three replicates generated for both the standards mixture and urine sample with CliqueMS. The three replicates were acquired using XCMS with various signal-to-noise thresholds (sn1-sn24), and the prefilters option was either enabled and set to its default values or entirely disabled (nf). Certain features were not consistently grouped in the same clique, when a specific dataset was analysed several times with identical parameters. As the CliqueMS scoring system is strongly dependent on connectivity, differences in the content of the pseudo-spectra can affect the annotation of the metabolites. In the end, several analytes were annotated in only one or two replicates.*

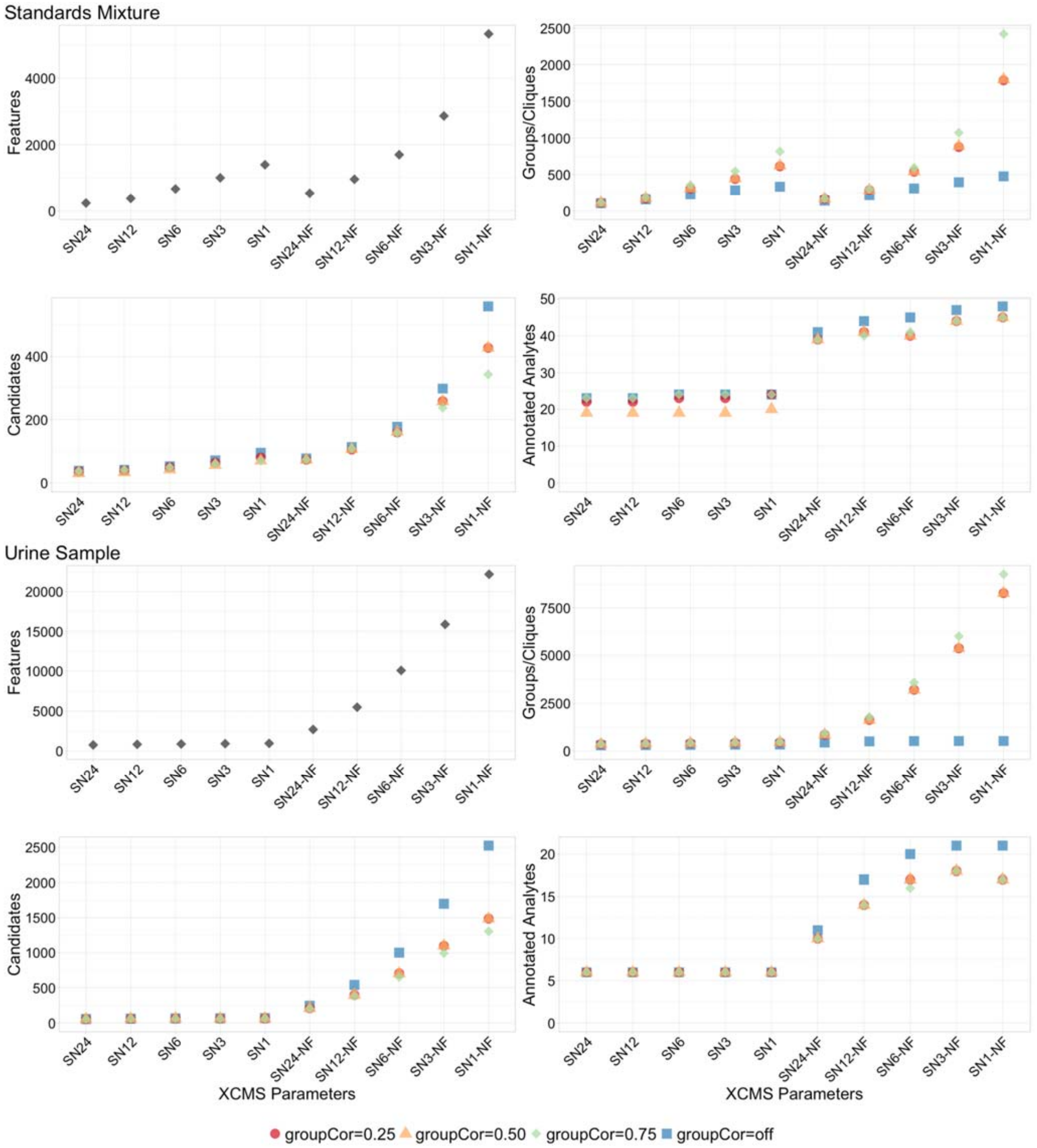

Figure S3: Summary of the analysis of CAMERA's features annotation and grouping performance for standards mixture (52 analytes) and the urine sample (35 target analytes).
The tool was tested with different parameters, including different thresholds for the peak grouping function based on EICs shape similarity (groupCor). Lowering the threshold increased the tool's performances, as more analyte-derived features are grouped in the same spectrum, which consequently decreases the total number of pseudo-spectra, increases the number of annotated $[M+H]^+$ candidates and thus the amount of correctly annotated analytes.

Supplementary Tables:

Table S1: Summary of the 408 analytes producing [M+H]+ ions by flow injection analysis (UNIGE-FIA/MS). Analytes in yellow are the 6 analytes where mzAdan to failed to identify the $[M+H]^+$.

Name	Formula	Exact mass
3-Hydroxybutyric acid	C4H8O3	104.0473
Allantoin	C4H6N4O3	158.044
Diaminopimelic acid	C7H14N2O4	190.0954
Alpha-Linolenic acid	C18H30O2	278.2246
Lansoprazole	C16H14F3N3O2S	369.0759
NADH	C21H29N7O14P2	665.1248
Methylguanidine	C2H7N3	73.064
Glycine	C2H5NO2	75.032
Trimethylamine N-oxide	C3H9NO	75.0684
2-Pyrrolidinone	C4H7NO	85.0528
Gamma-Butyrolactone	C4H6O2	86.0368
Beta-Alanine	C3H7NO2	89.0477
Sarcosine	C3H7NO2	89.0477
L-Alanine	C3H7NO2	89.0477
D-Alanine	C3H7NO2	89.0477
2-Piperidinone	C5H9NO	99.0684
Senecioic acid	C5H8O2	100.0524
L-Alpha-aminobutyric acid	C4H9NO2	103.0633
D-Alpha-aminobutyric acid	C4H9NO2	103.0633
Gamma-Aminobutyric acid	C4H9NO2	103.0633
Dimethylglycine	C4H9NO2	103.0633
3-Aminoisobutanoic acid	C4H9NO2	103.0633
2,3-Diaminopropionic acid	C3H8N2O2	104.0586
L-Serine	C3H7NO3	105.0426
Diethanolamine	C4H11NO2	105.079
Hypotaurine	C2H7NO2S	109.0197
Cytosine	C4H5N3O	111.0433
Histamine	C5H9N3	111.0796
Uracil	C4H4N2O2	112.0273
Creatinine	C4H7N3O	113.0589
Dihydrouracil	C4H6N2O2	114.0429
N-Methylhydantoin	C4H6N2O2	114.0429
Delta-Hexanolactone	C6H10O2	114.0681
Gamma-Caprolactone	C6H10O2	114.0681
L-Proline	C5H9NO2	115.0633
Levulinic acid	C5H8O3	116.0473
Acetylglycine	C4H7NO3	117.0426

Betaine	C5H11NO2	117.079
L-Valine	C5H11NO2	117.079
5-Aminopentanoic acid	C5H11NO2	117.079
N-Methyl-a-aminoisobutyric acid	C5H11NO2	117.079
L-Homoserine	C4H9NO3	119.0582
L-Threonine	C4H9NO3	119.0582
Purine	C5H4N4	120.0436
L-Cysteine	C3H7NO2S	121.0197
Benzamide	C7H7NO	121.0528
Niacinamide	C6H6N2O	122.048
Nicotinic acid	C6H5NO2	123.032
Picolinic acid	C6H5NO2	123.032
4-Methylcatechol	C7H8O2	124.0524
Taurine	C2H7NO3S	125.0147
Thymine	C5H6N2O2	126.0429
Imidazoleacetic acid	C5H6N2O2	126.0429
2,4-Diamino-6-hydroxypyrimidine	C4H6N4O	126.0542
Pyroglutamic acid	C5H7NO3	129.0426
Pyrrolidonecarboxylic acid	C5H7NO3	129.0426
Pipecolic acid	C6H11NO2	129.079
1,1-Dimethylbiguanide	C4H11N5	129.1014
N-Acetylputrescine	C6H14N2O	130.1106
4-Hydroxyproline	C5H9NO3	131.0582
N-Acetyl-L-alanine	C5H9NO3	131.0582
5-Aminolevulinic acid	C5H9NO3	131.0582
Creatine	C4H9N3O2	131.0695
3-Methylindole	C9H9N	131.0735
L-Norleucine	C6H13NO2	131.0946
L-Alloisoleucine	C6H13NO2	131.0946
L-Leucine	C6H13NO2	131.0946
L-Isoleucine	C6H13NO2	131.0946
Aminocaproic acid	C6H13NO2	131.0946
Beta-Leucine	C6H13NO2	131.0946
Monoethyl malonic acid	C5H8O4	132.0423
Methylsuccinic acid	C5H8O4	132.0423
L-Asparagine	C4H8N2O3	132.0535
Glycyl-glycine	C4H8N2O3	132.0535
Ornithine	C5H12N2O2	132.0899
Indoxyl	C8H7NO	133.0528
Methylcysteine	C4H9NO2S	135.0354
Adenine	C5H5N5	135.0545
N-Methylnicotinamide	C7H8N2O	136.0637
p-Aminobenzoic acid	C7H7NO2	137.0477
m-Aminobenzoic acid	C7H7NO2	137.0477

Trigonelline	C7H7NO2	137.0477
3-Pyridylacetic acid	C7H7NO2	137.0477
2-Aminobenzoic acid	C7H7NO2	137.0477
Tyramine	C8H11NO	137.0841
2-Hydroxyphenethylamine	C8H11NO	137.0841
Urocanic acid	C6H6N2O2	138.0429
6-Hydroxynicotinic acid	C6H5NO3	139.0269
Phosphonoacetate	C2H5O5P	139.9875
1,3-Dimethyluracil	C6H8N2O2	140.0586
L-Histidinol	C6H11N3O	141.0902
3-Hexenedioic acid	C6H8O4	144.0423
4-Hydroxycyclohexyl-carboxylic acid	C7H12O3	144.0786
4-Guanidinobutanoic acid	C5H11N3O2	145.0851
Monomethyl glutaric acid	C6H10O4	146.0579
Methylglutaric acid	C6H10O4	146.0579
2,2-Dimethylsuccinic acid	C6H10O4	146.0579
L-Glutamine	C5H10N2O3	146.0691
D-Glutamine	C5H10N2O3	146.0691
L-Lysine	C6H14N2O2	146.1055
D-Lysine	C6H14N2O2	146.1055
L-Glutamic acid	C5H9NO4	147.0532
Ribonolactone	C5H8O5	148.0372
trans-Cinnamic acid	C9H8O2	148.0524
Cinnamic acid	C9H8O2	148.0524
L-Methionine	C5H11NO2S	149.051
3-Methyladenine	C6H7N5	149.0701
6-Methyladenine	C6H7N5	149.0701
4-Ethylbenzoic acid	C9H10O2	150.0681
(+)-(S)-Carvone	C10H14O	150.1045
Acetaminophen	C8H9NO2	151.0633
2-Phenylglycine	C8H9NO2	151.0633
Phenylpropanolamine	C9H13NO	151.0997
4-Hydroxy-3-methylbenzoic acid	C8H8O3	152.0473
3-Cresotinic acid	C8H8O3	152.0473
p-Anisic acid	C8H8O3	152.0473
D-Arabitol	C5H12O5	152.0685
L-Arabitol	C5H12O5	152.0685
Ribitol	C5H12O5	152.0685
D-Xylitol	C5H12O5	152.0685
3-Aminosalicylic acid	C7H7NO3	153.0426
FAPy-adenine	C5H7N5O	153.0651
Dopamine	C8H11NO2	153.079
2,6-Dihydroxybenzoic acid	C7H6O4	154.0266

2,4-Dihydroxybenzoic acid	C7H6O4	154.0266
L-Histidine	C6H9N3O2	155.0695
2-Chlorobenzoic acid	C7H5ClO2	155.9978
L-Dihydroorotic acid	C5H6N2O4	158.0328
Succinylacetone	C7H10O4	158.0579
Isovalerylglycine	C7H13NO3	159.0895
DL-2-Aminooctanoic acid	C8H17NO2	159.1259
3-Methyladipic acid	C7H12O4	160.0736
Pimelic acid	C7H12O4	160.0736
Tryptamine	C10H12N2	160.1
Indole-3-carboxylic acid	C9H7NO2	161.0477
Aminoadipic acid	C6H11NO4	161.0688
Tryptophanol	C10H11NO	161.0841
L-Carnitine	C7H15NO3	161.1052
5-Hydroxylysine	C6H14N2O3	162.1004
Acetylcysteine	C5H9NO3S	163.0303
6-Dimethylaminopurine	C7H9N5	163.0858
2-Phenylbutyric acid	C10H12O2	164.0837
Isoeugenol	C10H12O2	164.0837
Methionine sulfoxide	C5H11NO3S	165.046
1-Methylguanine	C6H7N5O	165.0651
7-Methylguanine	C6H7N5O	165.0651
L-Phenylalanine	C9H11NO2	165.079
Benzocaine	C9H11NO2	165.079
Phthalic acid	C8H6O4	166.0266
7-Methylxanthine	C6H6N4O2	166.0491
3-Phenoxypropionic acid	C9H10O3	166.063
Perillic acid	C10H14O2	166.0994
Quinolinic acid	C7H5NO4	167.0219
3-Methoxytyramine	C9H13NO2	167.0946
Phenylephrine	C9H13NO2	167.0946
Phosphoenolpyruvic acid	C3H5O6P	167.9824
Homogentisic acid	C8H8O4	168.0423
5-Methoxysalicylic acid	C8H8O4	168.0423
Vanillic acid	C8H8O4	168.0423
Pyridoxamine	C8H12N2O2	168.0899
2-Amino-3-phosphonopropionic acid	C3H8NO5P	169.014
2-Furoylglycine	C7H7NO4	169.0375
6-Hydroxydopamine	C8H11NO3	169.0739
Pyridoxine	C8H11NO3	169.0739
3-Methylhistidine	C7H11N3O2	169.0851
1-Methylhistidine	C7H11N3O2	169.0851
Diethylthiophosphate	C4H11O3PS	170.0167
Glycerol 3-phosphate	C3H9O6P	172.0137

Menadione	C11H8O2	172.0524
Glycylproline	C7H12N2O3	172.0848
Quinaldic acid	C10H7NO2	173.0477
Suberic acid	C8H14O4	174.0892
L-Arginine	C6H14N4O2	174.1117
D-Arginine	C6H14N4O2	174.1117
Guanidinosuccinic acid	C5H9N3O4	175.0593
Indoleacetic acid	C10H9NO2	175.0633
Citrulline	C6H13N3O3	175.0957
Ascorbic acid	C6H8O6	176.0321
Ureidosuccinic acid	C5H8N2O5	176.0433
Allantoic acid	C4H8N4O4	176.0546
2-Isopropylmalic acid	C7H12O5	176.0685
Serotonin	C10H12N2O	176.095
Cotinine	C10H12N2O	176.095
N-Formyl-L-methionine	C6H11NO3S	177.046
Gluconolactone	C6H10O6	178.0477
4-Methoxycinnamic acid	C10H10O3	178.063
Isoxanthopterin	C6H5N5O2	179.0443
Hippuric acid	C9H9NO3	179.0582
Aspirin	C9H8O4	180.0423
Caffeic acid	C9H8O4	180.0423
Nicotinuric acid	C8H8N2O3	180.0535
D-Mannose	C6H12O6	180.0634
Scyllitol	C6H12O6	180.0634
Theobromine	C7H8N4O2	180.0647
3-Methoxybenzenepropanoic acid	C10H12O3	180.0786
L-Tyrosine	C9H11NO3	181.0739
o-Tyrosine	C9H11NO3	181.0739
9-Methyluric acid	C6H6N4O3	182.044
Homovanillic acid	C9H10O4	182.0579
Mannitol	C6H14O6	182.079
Galactitol	C6H14O6	182.079
Sorbitol	C6H14O6	182.079
L-Homocysteic acid	C4H9NO5S	183.0201
Normetanephrine	C9H13NO3	183.0895
Phosphoserine	C3H8NO6P	185.0089
3-Phosphoglyceric acid	C3H7O7P	185.9929
Indoleacrylic acid	C11H9NO2	187.0633
Azelaic acid	C9H16O4	188.1049
N-Alpha-acetyllysine	C8H16N2O3	188.1161
Glycyl-L-leucine	C8H16N2O3	188.1161
Homo-L-arginine	C7H16N4O2	188.1273
N-Acetylglutamic acid	C7H11NO5	189.0637

3-Indolepropionic acid	C11H11NO2	189.079
Homocitrulline	C7H15N3O3	189.1113
5-Methoxytryptamine	C11H14N2O	190.1106
5-Hydroxyindoleacetic acid	C10H9NO3	191.0582
N-Acetyl-L-methionine	C7H13NO3S	191.0616
5-Methoxytryptophol	C11H13NO2	191.0946
Citric acid	C6H8O7	192.027
Isocitric acid	C6H8O7	192.027
D-threo-Isocitric acid	C6H8O7	192.027
Quinic acid	C7H12O6	192.0634
Phenylacetylglycine	C10H11NO3	193.0739
Isoferulic acid	C10H10O4	194.0579
trans-Ferulic acid	C10H10O4	194.0579
4-Aminohippuric acid	C9H10N2O3	194.0691
Alpha-Hydroxyhippuric acid	C9H9NO4	195.0532
Gluconic acid	C6H12O7	196.0583
Galactonic acid	C6H12O7	196.0583
1,3-Dimethyluric acid	C7H8N4O3	196.0596
1,9-Dimethyluric acid	C7H8N4O3	196.0596
Guaifenesin	C10H14O4	198.0892
Sebacic acid	C10H18O4	202.1205
L-Acetylcarnitine	C9H17NO4	203.1158
L-Tryptophan	C11H12N2O2	204.0899
Lipoamide	C8H15NOS2	205.0595
Indolelactic acid	C11H11NO3	205.0739
(R)-lipoic acid	C8H14O2S2	206.0435
Ibuprofen	C13H18O2	206.1307
N-Acetyl-L-phenylalanine	C11H13NO3	207.0895
Chalcone	C15H12O	208.0888
1,3,7-Trimethyluric acid	C8H10N4O3	210.0753
Phosphocreatine	C4H10N3O5P	211.0358
3-Methoxytyrosine	C10H13NO4	211.0845
3-Chlorotyrosine	C9H10ClNO3	215.0349
Undecanedioic acid	C11H20O4	216.1362
3-Hydroxydodecanoic acid	C12H24O3	216.1725
12-Hydroxydodecanoic acid	C12H24O3	216.1725
5-Methoxydimethyltryptamine	C13H18N2O	218.1419
Pantothenic acid	C9H17NO5	219.1107
N-Acetylgalactosamine	C8H15NO6	221.0899
Beta-N-Acetylglucosamine	C8H15NO6	221.0899
N-Acetyl-D-glucosamine	C8H15NO6	221.0899
N-Acetylmannosamine	C8H15NO6	221.0899
L-Cystathionine	C7H14N2O4S	222.0674
Hydroxykynurenine	C10H12N2O4	224.0797

3-Nitrotyrosine	C9H10N2O5	226.059
Carnosine	C9H14N4O3	226.1066
Deoxycytidine	C9H13N3O4	227.0906
Deoxyuridine	C9H12N2O5	228.0746
Traumatic acid	C12H20O4	228.1362
D-Ribose 5-phosphate	C5H11O8P	230.0192
Naproxen	C14H14O3	230.0943
Dodecanedioic acid	C12H22O4	230.1518
Melatonin	C13H16N2O2	232.1212
5-Methoxytryptophan	C12H14N2O3	234.1004
3,4,5-Trimethoxycinnamic acid	C12H14O5	238.0841
Thymidine	C10H14N2O5	242.0903
Uridine	C9H12N2O6	244.0695
Biotin	C10H16N2O3S	244.0882
1,11-Undecanedicarboxylic acid	C13H24O4	244.1675
Pyridoxal 5'-phosphate	C8H10NO6P	247.0246
Deoxyinosine	C10H12N4O4	252.0859
Triamterene	C12H11N7	253.1076
Daidzein	C15H10O4	254.0579
Diphenhydramine	C17H21NO	255.1623
Glycerophosphocholine	C8H20NO6P	257.1028
Ribothymidine	C10H14N2O6	258.0852
Tetradecanedioic acid	C14H26O4	258.1831
Glucosamine 6-phosphate	C6H14NO8P	259.0457
Propranolol	C16H21NO2	259.1572
Mannose 6-phosphate	C6H13O9P	260.0297
Glucose 6-phosphate	C6H13O9P	260.0297
Fructose 6-phosphate	C6H13O9P	260.0297
Glucose 1-phosphate	C6H13O9P	260.0297
Alpha-Aspartyl-lysine	C10H19N3O5	261.1325
Atenolol	C14H22N2O3	266.163
Adenosine	C10H13N5O4	267.0968
Deoxyguanosine	C10H13N5O4	267.0968
Metoprolol	C15H25NO3	267.1834
Inosine	C10H12N4O5	268.0808
Genistein	C15H10O5	270.0528
Estrone	C18H22O2	270.162
Doxylamine	C17H22N2O	270.1732
Dextromethorphan	C18H25NO	271.1936
Estradiol	C18H24O2	272.1776
Phloretin	C15H14O5	274.0841
Ethenodeoxyadenosine	C12H13N5O3	275.1018
6-Phosphogluconic acid	C6H13O10P	276.0246
L-Aspartyl-L-phenylalanine	C13H16N2O5	280.1059

Imipramine	C19H24N2	280.1939
1-Methyladenosine	C11H15N5O4	281.1124
Guanosine	C10H13N5O5	283.0917
Xanthosine	C10H12N4O6	284.0757
Retinal	C20H28O	284.214
Kaempferol	C15H10O6	286.0477
Luteolin	C15H10O6	286.0477
Salicin	C13H18O7	286.1053
Androstenedione	C19H26O2	286.1933
Hexadecanedioic acid	C16H30O4	286.2144
16b-Hydroxyestradiol	C18H24O3	288.1725
Estriol	C18H24O3	288.1725
Androstanedione	C19H28O2	288.2089
Testosterone	C19H28O2	288.2089
Dehydroepiandrosterone	C19H28O2	288.2089
Epicatechin	C15H14O6	290.079
Argininosuccinic acid	C10H18N4O6	290.1226
Androsterone	C19H30O2	290.2246
Aspartame	C14H18N2O5	294.1216
17a-Ethynylestradiol	C20H24O2	296.1776
Hydrochlorothiazide	C7H8ClN3O4S2	296.9645
5'-Methylthioadenosine	C11H15N5O3S	297.0896
13-cis-Retinoic acid	C20H28O2	300.2089
All-trans-retinoic acid	C20H28O2	300.2089
Sphinganine	C18H39NO2	301.2981
Ellagic acid	C14H6O8	302.0063
Quercetin	C15H10O7	302.0427
2-Methoxyestradiol	C19H26O3	302.1882
Scopolamine	C17H21NO4	303.1471
Iodotyrosine	C9H10INO3	306.9705
dCMP	C9H14N3O7P	307.0569
Glutathione	C10H17N3O6S	307.0838
N-Acetylneuraminic acid	C11H19NO9	309.106
16-Dehydroprogesterone	C21H28O2	312.2089
Ranitidine	C13H22N4O3S	314.1413
Octadecanedioic acid	C18H34O4	314.2457
5-Thymidylic acid	C10H15N2O8P	322.0566
Cytidine monophosphate	C9H14N3O8P	323.0519
Uridine 5'-monophosphate	C9H13N2O9P	324.0359
Cyclic AMP	C10H12N5O6P	329.0525
Adenosine 2',3'-cyclic phosphate	C10H12N5O6P	329.0525
Furosemide	C12H11ClN2O5S	330.0077
11a-Hydroxyprogesterone	C21H30O3	330.2195
Deoxycorticosterone	C21H30O3	330.2195

17-Hydroxyprogesterone	C21H30O3	330.2195
Deoxyadenosine monophosphate	C10H14N5O6P	331.0682
Famotidine	C8H15N7O2S3	337.0449
Danazol	C22H27NO2	337.2042
Canrenone	C22H28O3	340.2038
Cellobiose	C12H22O11	342.1162
Alpha-Lactose	C12H22O11	342.1162
Sucrose	C12H22O11	342.1162
Melibiose	C12H22O11	342.1162
Lactulose	C12H22O11	342.1162
Isomaltose	C12H22O11	342.1162
Clotrimazole	C22H17ClN2	344.108
Maltitol	C12H24O11	344.1319
Medroxyprogesterone	C22H32O3	344.2351
Omeprazole	C17H19N3O3S	345.1147
Corticosterone	C21H30O4	346.2144
Cortexolone	C21H30O4	346.2144
2'-Deoxyguanosine 5'-monophosphate	C10H14N5O7P	347.0631
Adenosine monophosphate	C10H14N5O7P	347.0631
Inosinic acid	C10H13N4O8P	348.0471
Chlorogenic acid	C16H18O9	354.0951
2-Phenylaminoadenosine	C16H18N6O4	358.139
Cortisone	C21H28O5	360.1937
Levofloxacin	C18H20FN3O4	361.1438
Cortisol	C21H30O5	362.2093
Guanosine monophosphate	C10H14N5O8P	363.058
Biocytin	C16H28N4O4S	372.1831
Riboflavin	C17H20N4O6	376.1383
Loratadine	C22H23ClN2O2	382.1448
Vitamin D3	C27H44O	384.3392
Cholestenone	C27H44O	384.3392
5alpha-Cholestanone	C27H46O	386.3549
Nutriacholic acid	C24H38O4	390.277
7a-Hydroxy-3-oxo-5b-cholanoic acid	C24H38O4	390.277
Hyodeoxycholic acid	C24H40O4	392.2927
Chenodeoxycholic acid	C24H40O4	392.2927
Deoxycholic acid	C24H40O4	392.2927
Pregnenolone sulfate	C21H32O5S	396.197
7-Ketocholesterol	C27H44O2	400.3341
Uridine 5'-diphosphate	C9H14N2O12P2	404.0022
ADP	C10H15N5O10P2	427.0294
3,5-Diiodo-L-tyrosine	C9H9I2NO3	432.8672
Uvaol	C30H50O2	442.3811

Guanosine diphosphate	C10H15N5O11P2	443.0243
Verapamil	C27H38N2O4	454.2832
Flavin Mononucleotide	C17H21N4O9P	456.1046
Glycocholic acid	C26H43NO6	465.309
Glycyrrhetinic acid	C30H46O4	470.3396
Taurodeoxycholic acid	C26H45NO6S	499.2968
Maltotriose	C18H32O16	504.169
Raffinose	C18H32O16	504.169
dGTP	C10H16N5O13P3	506.9957
Adenosine triphosphate	C10H16N5O13P3	506.9957
Inosine triphosphate	C10H15N4O14P3	507.9798
Taurocholic acid	C26H45NO7S	515.2917
Guanosine triphosphate	C10H16N5O14P3	522.9907
Uridine diphosphate glucose	C15H24N2O17P2	566.055
Mesoporphyrin IX	C34H38N4O4	566.2893
Bilirubin	C33H36N4O6	584.2635
Hematoporphyrin IX	C34H38N4O6	598.2791
Hesperidin	C28H34O15	610.1898
Liothyronine	C15H12I3NO4	650.79
Coproporphyrin I	C36H38N4O8	654.269
PC(16:0/16:0)	C40H80NO8P	733.5622

Table S2: Summary of the 52 metabolites of the LC-mixture (UNIGE-LC/MS).

ID	Name	Formula	Mass (Da)	Conc. (µg/ml)	RT (min)
M1	L-Lysine	C6H14N2O2	146.106	100	1.12
M2	L-Histidine	C6H9N3O2	155.07	100	1.15
M3	Carnosine	C9H14N4O3	226.107	25	1.16
M4	1-Methylhistidine	C7H11N3O2	169.085	10	1.17
M5	Diethanolamine	C4H11NO2	105.079	25	1.2
M6	Glycerophosphocholine	C8H20NO6P	257.103	20	1.2
M7	L-Glutamine	C5H10N2O3	146.069	100	1.21
M8	Homo-L-arginine	C7H16N4O2	188.127	20	1.22
M9	Betaine	C5H11NO2	117.079	20	1.25
M10	N-Methylnicotinamide	C7H8N2O	136.064	10	1.26
M11	L-Carnitine	C7H15NO3	161.105	5	1.27
M12	N-Acetylneuraminic_acid	C11H19NO9	309.106	100	1.27
M13	Trigonelline	C7H7NO2	137.048	10	1.3
M14	Creatinine	C4H7N3O	113.059	20	1.31
M15	Creatine	C4H9N3O2	131.07	25	1.34
M16	L-Proline	C5H9NO2	115.063	100	1.35
M17	Homocitrulline	C7H15N3O3	189.111	25	1.38
M18	N-Acetylputrescine	C6H14N2O	130.1110	20	1.56
M19	L-Acetylcarnitine	C9H17NO4	203.116	5	1.82
M20	4-Guanidinobutanoic_acid	C5H11N3O2	145.085	20	1.88
M21	3-Methyladenine	C6H7N5	149.07	5	1.98
M22	Urocanic_acid	C6H6N2O2	138.043	100	2.08
M23	1-Methyladenosine	C11H15N5O4	281.112	20	2.37
M24	7-Methylguanine	C6H7N5O	165.065	20	2.94
M25	Tyramine	C8H11NO	137.084	100	3.33
M26	Cotinine	C10H12N2O	176.095	5	3.86
M27	Guanosine	C10H13N5O5	283.092	100	4
M28	Cyclic_AMP	C10H12N5O6P	329.053	100	4.25
M29	Ethenodeoxyadenosine	C12H13N5O3	275.1020	20	4.58
M30	3-Chlorotyrosine	C9H10ClNO3	215.035	5	4.83
M31	1,9-Dimethyluric_acid	C7H8N4O3	196.06	100	5.34
M32	Pantothenic_acid	C9H17NO5	219.111	20	5.7
M33	Acetaminophen	C8H9NO2	151.063	20	5.75
M34	1,3-Dimethyluric_acid	C7H8N4O3	196.06	100	5.79
M35	Theobromine	C7H8N4O2	180.065	20	5.93
M36	Isovalerylglycine	C7H13NO3	159.09	25	7.01
M37	L-Aspartyl-L-phenylalanine	C13H16N2O5	280.1060	25	7.06
M38	Monomethyl_glutaric_acid	C6H10O4	146.0580	100	7.57
M39	1,3,7-Trimethyluric_acid	C8H10N4O3	210.075	20	7.59

M40	Chlorogenic_acid	C16H18O9	354.095	100	7.62
M41	Hippuric_acid	C9H9NO3	179.058	25	7.67
M42	5'-Methylthioadenosine	C11H15N5O3S	297.09	20	7.68
M43	Quinaldic_acid	C10H7NO2	173.048	20	7.93
M44	Phenylacetylglycine	C10H11NO3	193.074	20	8.53
M45	Riboflavin	C17H20N4O6	376.138	20	9.55
M46	N-Acetyl-L-phenylalanine	C11H13NO3	207.09	20	10.36
M47	Indoleacetic_acid	C10H9NO2	175.063	25	11.38
M48	Azelaic_acid	C9H16O4	188.105	25	12.86
M49	Phloretin	C15H14O5	274.084	100	14.23
M50	Cortisone	C21H28O5	360.194	10	14.43
M51	Taurocholic_acid	C26H45NO7S	515.292	100	17.36
M52	Glycocholic_acid	C26H43NO6	465.309	25	18.34

Table S3: Summary of the 35 metabolites considered in the human urine sample.

ID	Name	Formula	Mass (Da)	Int (cps)	RT (min)
U1	L-Histidine	C6H9N3O2	155.069	9.85E+04	1.2
U2	3-Methylhistidine	C7H11N3O2	169.085	5.32E+05	1.21
U3	Betaine	C5H11NO2	117.079	1.04E+05	1.27
U4	Alpha-aminobutyric acid	C4H9NO2	103.063	3.65E+03	1.27
U5	N-Methylnicotinamide	C7H8N2O	136.064	1.11E+05	1.28
U6	L-Carnitine	C7H15NO3	161.105	5.37E+05	1.29
U7	Trigonelline	C7H7NO2	137.048	2.81E+05	1.31
U8	Creatinine	C4H7N3O	113.059	1.07E+06	1.35
U9	Glycylproline	C7H12N2O3	172.085	1.91E+04	1.68
U10	L-Acetylcarnitine	C9H17NO4	203.116	1.16E+06	1.82
U11	Urocanic acid	C6H6N2O2	138.043	6.87E+04	2
U12	Pyroglutamic acid	C5H7NO3	129.043	1.36E+05	2.11
U13	3-Methyladenine	C6H7N5	149.07	9.67E+04	2.2
U14	L-Tyrosine	C9H11NO3	181.074	8.71E+04	2.62
U15	7-Methylguanine	C6H7N5O	165.065	3.65E+05	2.76
U16	L-Leucine	C6H13NO2	131.095	1.13E+04	3.13
U17	Cotinine	C10H12N2O	176.095	1.50E+05	3.74
U18	Cyclic AMP	C10H12N5O6P	329.053	3.87E+04	3.97
U19	Adenosine	C10H13N5O4	267.097	9.90E+04	4.09
U20	1,9-Dimethyluric acid	C7H8N4O3	196.06	8.91E+03	4.63
U21	L-Phenylalanine	C9H11NO2	165.079	9.47E+04	4.72
U22	2-Furoylglycine	C7H7NO4	169.038	1.94E+05	4.83
U23	2-Piperidinone	C5H9NO	99.068	8.66E+04	5.08
U24	Pantothenic acid	C9H17NO5	219.111	1.66E+05	5.39
U25	1,3-Dimethyluric acid	C7H8N4O3	196.06	3.27E+05	5.42
U26	Theobromine	C7H8N4O2	180.065	4.33E+05	5.55
U27	L-Tryptophan	C11H12N2O2	204.09	1.69E+05	6.14
U28	5-Hydroxyindoleacetic acid	C10H9NO3	191.058	5.65E+04	6.78
U29	1,3,7-Trimethyluric acid	C8H10N4O3	210.075	5.82E+04	7.09
U30	5'-Methylthioadenosine	C11H15N5O3S	297.09	1.80E+04	7.24
U31	Hippuric acid	C9H9NO3	179.058	1.42E+06	7.25
U32	Indolelactic acid	C11H11NO3	205.074	2.19E+04	9.66
U33	Indoleacetic acid	C10H9NO2	175.063	7.43E+04	10.59
U34	Cortisone	C21H28O5	360.194	3.14E+04	13.24
U35	Androsterone	C19H30O2	290.225	1.75E+04	16.07

Table S4: Annotation set used for the manual annotation of the standards mixture mass spectra, including PeakView data and XCMS pseudo-spectra. Mass differences are relative to the Molecular Weight.

Adduct or Loss	mass diff
$[M+H-H_2O]^+$	-17.003
$[M+H-NH_3]^+$	-16.019
$[M+H]^+$	1.007
$[2M+H]^+$	1.007
$[3M+H]^+$	1.007
$[M+NH_4]^+$	18.034
$[M+Na]^+$	22.989
$[2M+Na]^+$	22.989
$[2M+K]^+$	38.963
$[M+K]^+$	38.963
$[M-H+2Na]^+$	44.971
$[M+Na+K-H]^+$	60.945
$[M+2K-H]^+$	76.919

Table S5: Default and optimised XCMS parameter settings obtained with IPO.

Parameters	Default	IPO 1	IPO 2
min_peakwidth:	5	14.5	7.3
max_peakwidth:	20	30	35
ppm:	15	42.5	35
mzdiff:	10	0.0155	-0.001
snthresh:	6	53.65	1
noise:	0	0	0
prefilter:	3	3	4
value_of_prefilter:	100	100	1

Table S6: Detailed parameters used for XCMS and CAMERA for the comparative analysis of XCMS-based annotation tools.

Tool	Functions	Arguments	Values
XCMS	xcmsSet	method	centwave
		polarity	positive
		ppm	15
		snthresh	1/3/6/12/24
		peakwidth	c(5,20)
		prefilter	c(3,100)/c(0,0)
CAMERA	groupFWHM	perfwhm	0.6
	groupCorr	cor_eic_th	0.75
	findIsotopes	mzabs	0.01
	findAdducts	rules	custom
		polarity	positive
		mzabs	0.01

Table S7: Detailed parameters used for XCMS and CliqueMS for the comparative analysis of XCMS-based annotation tools.

Tool	Functions	Arguments	Values
XCMS	findChromPeaks	method	centwave
		polarity	positive
		ppm	15
		snthresh	1/3/6/12/24
		peakwidth	c(5,20)
		prefilter	c(3,100)/c(0,0)
CLIQUEMS	getCliques	filter	TRUE
	getIsotopes	ppm	10
	getAnnotation	adinfo	custom
		polarity	positive
		ppm	10

Table S8: Detailed parameters used for XCMS, CAMERA and findMAIN for the comparative analysis of XCMS-based annotation tools.

Tool	Functions	Arguments	Values
XCMS	xcmsSet	method	centwave
		polarity	positive
		ppm	15
		snthresh	1/3/6/12/24
		peakwidth	c(5,20)
		prefilter	c(3,100)/c(0,0)
CAMERA	groupFWHM	perfwhm	0.6
FINDMAIN	findMAIN	adducthyp	[M+H]+
		rules	custom
		mzabs	0.01

Table S9: Detailed parameters used for XCMS, CAMERA and mzAdan for the comparative analysis of XCMS-based annotation tools.

Tool	Functions	Arguments	Values
XCMS	xcmsSet	method	centwave
		polarity	positive
		ppm	15
		snthresh	1/3/6/12/24
		peakwidth	c(5,20)
		prefilter	c(3,100)/c(0,0)
CAMERA	groupFWHM	perfwhm	0.6
MZADAN	parameters	int thresh.	disabled
		m/z thresh.	disabled
		deisotoping	1-100
		validation	disabled
	annotations	adducts	default
		neutrals	default
		isotopes	disabled
		multimers	3
	candidates	candidate connectivity	0

Table S10: Annotation set used with CAMERA for the comparative analysis of XCMS-based annotation tools.

name	nmol	charge	massdiff	oidscore	quasi	ips
$[M+H-H_2O]^+$	1	1	-17.0033	24	1	1
$[M+H-NH_3]^+$	1	1	-16.0193	16	1	1
$[M+H]^+$	1	1	1.0073	1	1	1
$[2M+H]^+$	2	1	1.0073	1	0	0.5
$[3M+H]^+$	3	1	1.0073	1	0	0.25
$[M+NH_4]^+$	1	1	18.0338	16	1	1
$[M+Na]^+$	1	1	22.9892	8	1	1
$[2M+Na]^+$	2	1	22.9892	8	0	0.5
$[M+K]^+$	1	1	38.9632	10	1	1
$[2M+K]^+$	2	1	38.9632	10	0	0.5
$[M+2Na-H]^+$	1	1	44.9712	34	0	0.5
$[M+2K-H]^+$	1	1	76.9190	60	0	0.5

Table S11: Annotation set used with CliqueMS for the comparative analysis of XCMS-based annotation tools.

adduct	log10freq	massdiff	nummol	charge
$[M+H-H_2O]^+$	-0.747608492	-17.003	1	1
$[M+H-NH_3]^+$	-1.588625136	-16.019	1	1
$[M+H]^+$	-0.268998652	1.007	1	1
$[2M+H]^+$	-1.223984816	1.007	2	1
$[3M+H]^+$	-2.269866373	1.007	3	1
$[M+NH_4]^+$	-2.491715123	18.034	1	1
$[M+Na]^+$	-0.963288183	22.989	1	1
$[2M+Na]^+$	-2.968836378	22.989	2	1
$[2M+K]^+$	-3.813934418	38.963	2	1
$[M+K]^+$	-2.415994409	38.963	1	1
$[M-H+2Na]^+$	-1.859691908	44.971	1	1
$[M-H+2K]^+$	-3.114964413	76.919	1	1

Table S12: Annotation set used with FindMAIN for the comparative analysis of XCMS-based annotation tools.

name
$[M+H-H_2O]^+$
$[M+H-NH^3]^+$
$[M+H]^+$
$[M+NH_4]^+$
$[M+Na]^+$
$[M+K]^+$
$[M-H+2Na]^+$
$[M-H+2K]^+$
$[2M+H]^+$
$[2M+K]^+$
$[2M+Na]^+$
$[3M+H]^+$

Table S13: Annotation set used with mzAdan for the comparative analysis of XCMS-based annotation tools.

name	value
NH_4-H	17.0266
Na-H	21.9820
K-H	37.9559
$-NH_3$	-17.0266
$-H_2O$	-18.0106
2M	NA
3M	NA

Table S14: Candidate lists proposed by mzAdan for the data in Figures 2 and 3

1) Candidate list for LC-MS for the RT=1.35 min

id	mass	m/z	int (cps)	int (%)	connectivity	cgc	cic	ccc
C0	115.0638	116.0711	54204	100	2	5	41.57	26.32
C1	131.0697	132.077	22089	40.8	2	7	37.39	36.84
C2	189.1122	190.1195	14690	27.1	3	6	20.16	31.58
C2	172.0849	173.0922	2046	3.8	1	6	20.16	31.58
C3	69.0575	70.0647	1266	2.3	0	1	0.88	5.26

2) Candidate list for flow injection analysis of L-proline

id	mass	m/z	int (cps)	int (%)	connectivity	cgc	cic	ccc
C0	115.0628	116.0701	51394	100	1	13	61.12	39.39
C1	69.0577	70.0649	18760	36.5	0	1	14.54	3.03
C2	370.0926	371.0999	9943	19.4	1	2	8.13	6.06
C2	387.1186	388.1259	543	1.1	1	2	8.13	6.06
C3	78.0136	79.0209	6058	11.8	0	1	4.7	3.03
C4	498.1976	499.2049	813	1.6	1	4	2.81	12.12
C5	231.0311	232.0384	1708	3.3	1	2	2.32	6.06
C6	199.0147	200.0219	1537	3	1	2	1.68	6.06
C7	234.1608	235.1681	1613	3.1	0	1	1.25	3.03
C8	354.0606	355.0679	979	1.9	0	1	0.76	3.03
C9	337.3326	338.3399	774	1.5	0	1	0.6	3.03
C10	321.0001	322.0073	572	1.1	0	1	0.44	3.03
C11	268.0729	269.0802	546	1.1	0	1	0.42	3.03
C12	147.9943	149.0016	544	1.1	0	1	0.42	3.03
C13	119.9995	121.0068	525	1	0	1	0.41	3.03
C14	527.101	528.1083	515	1	0	1	0.4	3.03

Table S15: Peak annotation summary for L-proline by FIA-MS analysis (Figure 3)

m/z	intensity	annotation	exact mas	error mmu
116.0701	51394	$[M+H]^+$	116.0706	-0.6
138.0521	7142	$[M+Na]^+$	138.0526	-0.5
160.0336	5423	$[M+2Na-H]^+$	160.0345	-0.9
253.1148	3536	$[2M+Na]^+$	253.1159	-1.1
275.0964	1564	$[2M+2Na-H]^+$	275.0978	-1.4
297.0783	1476	$[2M+3Na-2H]^+$	297.0798	-1.4
368.1772	1576	$[3M+Na]^+$	368.1792	-2
384.1424	1259	$[3M+K]^+$	384.1532	-10.7
		$[3M+Ca-H]^+$	384.1442	-1.8
390.1589	1001	$[3M+2Na-H]^+$	390.1612	-2.3
406.1240	1665	$[3M+Na+K-H]^+$	406.1351	-11.1
		$[3M+Na+Ca-2H]^+$	406.1262	-2.2
412.1404	702	$[3M+3Na-2H]^+$	412.1431	-2.7
428.1057	1048	$[3M+2Na+K-2H]^+$	428.1170	-11.3
		$[3M+2Na+Ca-3H]^+$	428.1081	-2.4
434.1223	1057	$[3M+4Na-3H]^+$	434.1250	-2.8
499.2049	813	$[4M+Ca-H]^+$	499.2075	-2.6
521.1866	1277	$[4M+Na+Ca-2H]^+$	521.1895	-2.9
543.1685	905	$[4M+2Na+Ca-3H]^+$	543.1714	-2.9
565.1498	626	$[4M+3Na+Ca-4H]^+$	565.1534	-3.6

Table S16: Relative intensities of the 13 adducts (including protonated forms) of the LC/MS analysis of the 52 analyte mix. Mass spectra were generated with PeakView (average at FWHM and background subtracted). Only annotations with correct retention times were considered (mzAdan and XIC).

ID	RT (min)	$[M+H-H_2O]^+$	$[M+H-NH_3]^+$	$[M+H]^+$	$[M+NH_4]^+$	$[M+Na]^+$	$[M+2Na-H]^+$	$[M+Na+K-H]^+$	$[M+K]^+$	$[M+2K-H]^+$	$[2M+H]^+$	$[2M+Na]^+$	$[2M+K]^+$	$[3M+H]^+$
M1	1.12	0	27.1	100	0	0	0	6.5	10.2	4.4	0	0	0	0
M2	1.15	0	0	100	0	11.1	1.9	5.9	10.5	5.8	0	0	0	0
M3	1.16	0	1.6	45.8	0	3.1	0	0	7.8	1.4	0	0	0	0
M4	1.17	0	0	94.1	0	3	0	0	4.9	0	0	0	0	0
M5	1.19	0	0	26.4	0	0	0	0	0	0	0	0	0	0
M6	1.20	0	0	100	0	0	0	0	11.5	0	0	0	0	0
M7	1.21	0	20.7	38	0	1.1	0	0	5.8	2.6	0	0	0	0
M8	1.22	0	0	19.5	0	0	0	0	1.7	0	0	0	0	0
M9	1.25	0	0	100	0	0	0	0	9	0	5.4	0	0	0
M10	1.26	0	0	33.5	0	0	0	0	0	0	0	0	0	0
M11	1.27	0	0	32.7	0	0	0	0	0	0	0	0	0	0
M12	1.27	5.1	0	19.7	0	0	0	0	5.9	0	0	0	0	0
M13	1.30	0	0	100	0	0	0	0	8.5	0	0	0	0	0
M14	1.31	0	0	61.4	0	1.7	0	0	15.4	0	13.7	1.3	1.9	0
M15	1.34	0	0	42.2	0	0	0	0	6.3	0	0	0	0	0
M16	1.35	0	0	100	0	0	0	0	5.1	2	2	0	0	0
M17	1.38	0	9.9	70	0	2.8	0	0	45.3	4.3	0	0	0	0
M18	1.56	0	8.5	100	0	0	0	0	1.9	0	0	0	0	0
M19	1.82	0	0	100	0	1.3	0	0	6	0	0	0	0	0
M20	1.88	0	0	100	0	0	0	0	2.6	0	0	0	0	0
M21	1.98	0	0	100	0	0	0	0	0	0	0	0	0	0
M22	2.08	1.6	0	100	0	0	0	0	0	0	0	0	0	0
M23	2.37	0	0	100	0	0	0	0	1.6	0	0	0	0	0
M24	2.94	0	0	100	0	3.7	0	0	25.7	1.9	3.6	0	0	0
M25	3.33	0	100	36.9	0	0	0	0	0	0	0	0	0	0
M26	3.86	0	0	100	0	0	0	0	6.8	0	0	0	0	0
M27	4.00	0	0	83.2	0	0	0	0	36.1	0	0	0	0	0
M28	4.25	0	0	100	0	0	0	0	11.5	2.2	0	0	0	0
M29	4.58	0	0	100	0	4.6	0	0	35.6	0	0	0	0	0
M30	4.83	0	0	100	0	0	0	0	0	0	0	0	0	0
M31	5.33	0	0	100	0	0	0	0	3.5	1.1	1.9	0	0	0
M32	5.70	0	0	92.2	0	7.1	0	0	0	0	0	0	0	0
M33	5.75	0	0	100	0	5.6	0	0	15.8	0	0	0	0	0
M34	5.79	0	0	100	0	0	0	0	0	0	0	0	0	0
M35	5.93	0	0	100	0	0	0	0	0	0	0	0	0	0
M36	7.01	0	0	100	0	12.2	0	2.5	40.7	12.2	0	0	0	0
M37	7.06	0	0	100	0	2.6	0	0	23.5	2.7	0	0	0	0

M38	7.57	100	0	20	0	6.3	0	1.5	9.7	6.1	0	0	0	0
M39	7.59	0	0	28.8	0	0	0	0	0	0	0	0	0	0
M40	7.62	0	0	100	3.5	2.8	0	0	40.8	0	0	0	0	0
M41	7.67	0	0	100	0	10.8	0	2	37.9	6.7	0	0	0	0
M42	7.68	0	0	13.9	0	0	0	0	0	0	0	0	0	0
M43	7.93	2.9	0	100	0	2.3	0	0	4.8	1.1	0	0	0	0
M44	8.53	0	0	100	0	6.8	0	1.9	51.2	6.5	0	0	0	0
M45	9.55	0	0	100	0	3	0	0	31.8	0	0	0	0	0
M46	10.36	7.5	0	100	0	9.5	0	2.4	51.6	7.8	0	0	0	0
M47	11.38	0	0	100	0	5.3	0	0	21.5	4.4	0	0	0	0
M48	12.86	67.2	0	62.4	85.1	34.2	0	5.1	100	9.3	0	0	0	0
M49	14.23	0	0	100	0	0	0	0	14.9	0	0	0	0	0
M50	14.43	0	0	100	0	3	0	0	24.6	0	0	0	0	0
M51	17.36	9.4	0	8.5	100	3.8	0	1.1	13.5	1.5	0	0	0	0
M52	18.34	16.3	0	100	95.6	13.4	0	0	27.8	0	0	0	0	0

Table S17: Relative intensities of the 13 adducts (including protonated forms) following manual annotation of the standards mixture mass spectra with features detected using XCMS with the recommended parameters, and pseudo-mass spectra were generated with CAMERA.

ID	RT (min)	[M+H-H20]+	[M+H-NH3]+	[M+H]+	[M+NH4]+	[M+Na]+	[M+2Na-H]+	[M+Na+K-H]+	[M+K]+	[M+2K-H]+	[2M+H]+	[2M+Na]+	[2M+K]+	[3M+H]+
M1	1.12	0	0	100	0	0	0	0	0	0	0	0	0	0
M2	1.15	0	0	100	0	0	0	0	0	0	0	0	0	0
M3	1.16	0	0	59	0	0	0	0	0	0	0	0	0	0
M4	1.17	0	0	100	0	0	0	0	0	0	0	0	0	0
M5	1.2	0	0	25.8	0	0	0	0	0	0	0	0	0	0
M6	1.2	0	0	100	0	0	0	0	14	0	0	0	0	0
M7	1.21	0	14.4	28.3	0	0	0	0	0	0	0	0	0	0
M8	1.22	0	0	100	0	0	0	0	0	0	0	0	0	0
M9	1.25	0	0	100	0	0	0	0	14.1	0	13.3	0	0	0
M10	1.26	0	0	46.5	0	0	0	0	0	0	0	0	0	0
M11	1.27	0	0	44.4	0	0	0	0	0	0	0	0	0	0
M12	1.27	0	0	100	0	0	0	0	33.9	0	0	0	0	0
M13	1.3	0	0	100	0	0	0	0	0	0	0	0	0	0
M14	1.31	0	0	80	0	0	0	0	21.1	0	21.5	0	0	0
M15	1.34	0	0	44.9	0	0	0	0	0	0	0	0	0	0
M16	1.35	0	0	100	0	0	0	0	0	0	0	0	0	0
M17	1.38	0	0	100	0	0	0	0	74.5	0	0	0	0	0
M18	1.56	0	81.1	100	0	0	0	0	0	0	0	0	0	0
M19	1.82	0	0	100	0	0	0	0	0	0	0	0	0	0
M20	1.88	0	0	100	0	0	0	0	0	0	0	0	0	0
M21	1.98	0	0	100	0	0	0	0	0	0	0	0	0	0
M22	2.08	0	0	100	0	0	0	0	0	0	0	0	0	0
M23	2.37	0	0	100	0	0	0	0	0	0	0	0	0	0
M24	2.94	0	0	100	0	0	0	0	31.2	0	0	0	0	0
M25	3.33	0	100	43.9	0	0	0	0	0	0	0	0	0	0
M26	3.86	0	0	100	0	0	0	0	0	0	0	0	0	0
M27	4	0	0	65.2	0	0	0	0	33.1	0	0	0	0	0
M28	4.25	0	0	100	0	0	0	0	0	0	0	0	0	0
M29	4.58	0	0	100	0	0	0	0	43.5	0	0	0	0	0
M30	4.83	0	0	0	0	0	0	0	0	0	0	0	0	0
M31	5.34	0	0	100	0	0	0	0	0	0	0	0	0	0
M32	5.7	0	0	100	0	0	0	0	67.7	0	0	0	0	0
M33	5.75	0	0	100	0	0	0	0	19.8	0	0	0	0	0
M34	5.79	0	0	100	0	0	0	0	0	0	0	0	0	0
M35	5.93	0	0	100	0	0	0	0	0	0	0	0	0	0
M36	7.01	0	0	100	0	0	0	0	52.6	0	0	0	0	0

M37	7.06	0	0	100	0	0	0	0	0	0	0	0	0	0
M38	7.57	100	0	23.7	0	0	0	0	0	0	0	0	0	0
M39	7.59	0	0	100	0	0	0	0	0	0	0	0	0	0
M40	7.62	0	0	100	0	0	0	0	48.8	0	0	0	0	0
M41	7.67	0	0	100	0	0	0	0	49.5	0	0	0	0	0
M42	7.68	0	0	0	0	0	0	0	0	0	0	0	0	0
M43	7.93	0	0	100	0	0	0	0	0	0	0	0	0	0
M44	8.53	0	0	100	0	0	0	0	76.5	0	0	0	0	0
M45	9.55	0	0	100	0	0	0	0	34.8	0	0	0	0	0
M46	10.36	0	0	100	0	0	0	0	58.9	0	0	0	0	0
M47	11.38	0	0	55	0	0	0	0	0	0	0	0	0	0
M48	12.86	47.3	0	42.2	55.2	24.9	0	0	74.3	0	0	0	0	0
M49	14.23	0	0	43.9	0	0	0	0	0	0	0	0	0	0
M50	14.43	0	0	100	0	0	0	0	28.6	0	0	0	0	0
M51	17.36	9.7	0	9.9	100	4.8	0	0	15.8	0	0	0	0	0
M52	18.34	0	0	100	98.6	0	0	0	32.7	0	0	0	0	0

Table S18: Number of features corresponding to known analytes or adducts detected in the standards mixture with XCMS.
Different values for parameters such as m/z tolerance in ppm (t) or peakwidth (pw) were used; prefilters were either enabled and set to their default values or entirely disabled (nf).

Parameters	Analytes	Annotations
default	50	33
t10	50	33
t20	50	35
pw(5,15)	50	33
pw(5,25)	50	33
default-nf	52	162
t10-nf	52	162
t20-nf	52	161
pw(5,15-nf)	52	162
pw(5,25)-nf	52	161

Different values for parameters, such as signal-to-noise (sn1-sn24) and prefilters were used; the latter were either enabled and set to their default values or entirely disabled (nf).

Parameters	Analytes	Annotations
sn24	50	32
sn12	50	32
default	50	33
sn3	50	33
sn1	50	33
sn24-nf	52	93
sn12-nf	52	132
default-nf	52	161
sn3-nf	52	195
sn1-nf	52	214

Table S19: Peak picking results for the standard mixture using XCMS with various signal-to-noise thresholds (sn1-sn24), and prefilters either enabled and set to their default values or entirely disabled (nf).

Detected analytes are represented by a (1), while (0) signifies that the feature corresponding to the protonated form of the analyte was not found.

ID	RT (min)	SN24	SN12	SN6	SN3	SN1	SN24-NF	SN12-NF	SN6-NF	SN3-NF	SN1-NF
M1	1.12	1	1	1	1	1	1	1	1	1	1
M2	1.15	1	1	1	1	1	1	1	1	1	1
M3	1.16	1	1	1	1	1	1	1	1	1	1
M4	1.17	1	1	1	1	1	1	1	1	1	1
M5	1.2	1	1	1	1	1	1	1	1	1	1
M6	1.2	1	1	1	1	1	1	1	1	1	1
M7	1.21	1	1	1	1	1	1	1	1	1	1
M8	1.22	1	1	1	1	1	1	1	1	1	1
M9	1.25	1	1	1	1	1	1	1	1	1	1
M10	1.26	1	1	1	1	1	1	1	1	1	1
M11	1.27	1	1	1	1	1	1	1	1	1	1
M12	1.27	1	1	1	1	1	1	1	1	1	1
M13	1.3	1	1	1	1	1	1	1	1	1	1
M14	1.31	1	1	1	1	1	1	1	1	1	1
M15	1.34	1	1	1	1	1	1	1	1	1	1
M16	1.35	1	1	1	1	1	1	1	1	1	1
M17	1.38	1	1	1	1	1	1	1	1	1	1
M18	1.56	1	1	1	1	1	1	1	1	1	1
M19	1.82	1	1	1	1	1	1	1	1	1	1
M20	1.88	1	1	1	1	1	1	1	1	1	1
M21	1.98	1	1	1	1	1	1	1	1	1	1
M22	2.08	1	1	1	1	1	1	1	1	1	1
M23	2.37	1	1	1	1	1	1	1	1	1	1
M24	2.94	1	1	1	1	1	1	1	1	1	1
M25	3.33	1	1	1	1	1	1	1	1	1	1
M26	3.86	1	1	1	1	1	1	1	1	1	1
M27	4	1	1	1	1	1	1	1	1	1	1
M28	4.25	1	1	1	1	1	1	1	1	1	1
M29	4.58	1	1	1	1	1	1	1	1	1	1
M30	4.83	0	0	0	0	0	1	1	1	1	1
M31	5.34	1	1	1	1	1	1	1	1	1	1
M32	5.7	1	1	1	1	1	1	1	1	1	1
M33	5.75	1	1	1	1	1	1	1	1	1	1
M34	5.79	1	1	1	1	1	1	1	1	1	1
M35	5.93	1	1	1	1	1	1	1	1	1	1
M36	7.01	1	1	1	1	1	1	1	1	1	1
M37	7.06	1	1	1	1	1	1	1	1	1	1

M38	7.57	1	1	1	1	1	1	1	1	1	1
M39	7.59	1	1	1	1	1	1	1	1	1	1
M40	7.62	1	1	1	1	1	1	1	1	1	1
M41	7.67	1	1	1	1	1	1	1	1	1	1
M42	7.68	0	0	0	0	0	1	1	1	1	1
M43	7.93	1	1	1	1	1	1	1	1	1	1
M44	8.53	1	1	1	1	1	1	1	1	1	1
M45	9.55	1	1	1	1	1	1	1	1	1	1
M46	10.36	1	1	1	1	1	1	1	1	1	1
M47	11.38	1	1	1	1	1	1	1	1	1	1
M48	12.86	1	1	1	1	1	1	1	1	1	1
M49	14.23	1	1	1	1	1	1	1	1	1	1
M50	14.43	1	1	1	1	1	1	1	1	1	1
M51	17.36	1	1	1	1	1	1	1	1	1	1
M52	18.34	1	1	1	1	1	1	1	1	1	1

Table S20: Summary of the annotation of the standards mixture using XCMS-CAMERA with various signal-to-noise thresholds (sn1-sn24), and prefilters either enabled and set to their default values or entirely disabled (nf).

Correctly annotated analytes are represented by a (1), while (0) signifies that the compound was either not detected, not annotated or incorrectly annotated.

ID	RT (min)	SN24	SN12	SN6	SN3	SN1	SN24-NF	SN12-NF	SN6-NF	SN3-NF	SN1-NF
M1	1.12	0	0	0	0	0	1	1	1	1	1
M2	1.15	0	0	0	0	0	1	1	1	1	1
M3	1.16	0	0	0	0	0	1	1	1	1	1
M4	1.17	0	0	0	0	0	1	0	0	1	1
M5	1.2	0	0	0	0	0	0	0	0	1	1
M6	1.2	1	1	1	1	1	1	1	1	1	1
M7	1.21	1	1	1	1	1	1	1	1	1	0
M8	1.22	0	0	0	0	0	1	1	1	1	1
M9	1.25	1	1	1	1	1	1	1	1	1	1
M10	1.26	0	0	0	0	0	0	0	0	0	0
M11	1.27	0	0	0	0	0	0	0	0	0	0
M12	1.27	1	1	1	1	1	1	1	1	1	1
M13	1.3	0	0	0	0	0	0	0	0	0	0
M14	1.31	1	1	1	1	1	1	1	1	1	1
M15	1.34	0	0	0	0	0	0	0	0	0	0
M16	1.35	0	0	0	0	0	1	1	1	1	1
M17	1.38	1	1	1	1	1	1	1	1	1	1
M18	1.56	0	0	1	1	1	0	1	1	1	1
M19	1.82	0	0	0	0	0	1	1	1	1	1
M20	1.88	0	0	0	0	0	1	1	1	1	1
M21	1.98	0	0	0	0	0	0	0	1	1	1
M22	2.08	0	0	0	0	0	0	1	1	1	1
M23	2.37	0	0	0	0	0	1	1	1	1	1
M24	2.94	1	1	1	1	1	1	1	1	1	1
M25	3.33	1	1	1	1	1	1	1	0	0	0
M26	3.86	0	0	0	0	0	1	1	1	1	1
M27	4	1	1	1	1	1	1	1	1	1	1
M28	4.25	0	0	0	0	0	1	1	1	1	1
M29	4.58	1	1	1	1	1	1	1	1	1	1
M30	4.83	0	0	0	0	0	0	0	0	0	1
M31	5.34	0	0	0	0	0	1	1	1	1	1
M32	5.7	1	1	1	1	1	1	1	1	1	1
M33	5.75	1	1	1	1	1	1	1	1	1	1
M34	5.79	0	0	0	0	0	0	0	0	1	1
M35	5.93	0	0	0	0	0	0	0	1	1	1
M36	7.01	1	1	1	1	1	1	1	1	1	1
M37	7.06	0	0	0	0	0	1	1	1	1	1

M38	7.57	1	1	1	1	1	1	1	1	1	1
M39	7.59	0	0	0	0	0	0	0	0	0	1
M40	7.62	1	1	1	1	1	1	1	1	1	1
M41	7.67	1	1	1	1	1	1	1	1	1	1
M42	7.68	0	0	0	0	0	0	0	0	0	0
M43	7.93	0	0	0	0	0	1	1	1	1	1
M44	8.53	1	1	1	1	1	1	1	1	1	1
M45	9.55	1	1	1	1	1	1	1	1	1	1
M46	10.36	1	1	1	1	1	1	1	1	1	1
M47	11.38	0	0	0	0	0	1	1	1	1	1
M48	12.86	1	1	1	1	1	1	1	1	1	1
M49	14.23	0	0	0	0	0	1	1	1	1	1
M50	14.43	1	1	1	1	1	1	1	1	1	1
M51	17.36	1	1	1	1	1	1	1	1	1	1
M52	18.34	1	1	1	1	1	1	1	1	1	1

Table S21: Summary of the annotation of the standards mixture using XCMS-CliqueMS with various signal-to-noise thresholds (sn1-sn24), and prefilters either enabled and set to their default values or entirely disabled (nf).

Correctly annotated analytes are represented by a (1), while (0) signifies that the compound was either not detected, not annotated or incorrectly annotated.

ID	RT (min)	SN24	SN12	SN6	SN3	SN1	SN24-NF	SN12-NF	SN6-NF	SN3-NF	SN1-NF
M1	1.12	0	0	0	0	0	1	1	1	0	0
M2	1.15	0	0	0	0	0	1	1	1	1	1
M3	1.16	0	0	0	0	0	1	1	1	1	0
M4	1.17	0	0	0	0	0	1	1	0	1	1
M5	1.2	0	0	0	0	0	0	0	0	1	1
M6	1.2	1	1	1	1	1	1	1	1	1	1
M7	1.21	1	1	1	1	1	1	1	1	1	1
M8	1.22	0	0	0	0	0	1	1	1	1	1
M9	1.25	1	1	1	1	1	1	1	1	1	1
M10	1.26	0	0	0	0	0	0	0	0	0	0
M11	1.27	0	0	0	0	0	0	1	1	1	1
M12	1.27	1	1	1	1	1	1	1	1	1	1
M13	1.3	0	0	0	0	0	1	1	1	1	1
M14	1.31	1	1	1	1	1	1	1	1	1	1
M15	1.34	0	0	0	0	0	1	1	1	1	1
M16	1.35	0	0	0	0	0	1	1	1	1	1
M17	1.38	1	1	1	1	1	1	1	1	1	1
M18	1.56	0	0	1	1	1	0	1	1	1	1
M19	1.82	0	0	0	0	0	1	1	1	1	1
M20	1.88	0	0	0	0	0	1	1	1	1	1
M21	1.98	0	0	0	0	0	0	0	0	1	1
M22	2.08	0	0	0	0	0	0	1	1	1	1
M23	2.37	0	0	0	0	0	1	1	1	1	1
M24	2.94	1	1	1	1	1	1	1	1	1	1
M25	3.33	1	1	1	1	1	1	1	1	1	1
M26	3.86	0	0	0	0	0	1	1	1	1	1
M27	4	1	1	1	1	1	1	1	1	1	1
M28	4.25	0	0	0	0	0	1	1	1	1	1
M29	4.58	1	1	1	1	1	1	1	1	1	1
M30	4.83	0	0	0	0	0	0	0	0	0	1
M31	5.34	0	0	0	0	0	1	1	1	1	1
M32	5.7	1	1	1	1	1	1	1	1	1	1
M33	5.75	1	1	1	1	1	1	1	1	1	1
M34	5.79	0	0	0	0	0	0	0	0	1	0
M35	5.93	0	0	0	0	0	0	0	1	1	1
M36	7.01	1	1	1	1	1	1	1	1	1	1
M37	7.06	0	0	0	0	0	1	1	1	1	1

M38	7.57	1	1	1	1	1	1	1	1	1	1
M39	7.59	0	0	0	0	0	0	0	0	0	1
M40	7.62	1	1	1	1	1	1	1	1	1	1
M41	7.67	1	1	1	1	1	1	1	1	1	1
M42	7.68	0	0	0	0	0	0	0	0	0	0
M43	7.93	0	0	0	0	0	1	1	1	1	1
M44	8.53	1	1	1	1	1	1	1	1	1	1
M45	9.55	1	1	1	1	1	1	1	1	1	1
M46	10.36	1	1	1	1	1	1	1	1	1	1
M47	11.38	0	0	0	0	0	1	1	1	1	1
M48	12.86	1	1	1	1	1	1	1	1	1	1
M49	14.23	0	0	0	0	0	1	1	1	1	1
M50	14.43	1	1	1	1	1	1	1	1	1	1
M51	17.36	1	1	1	1	1	1	1	1	1	1
M52	18.34	1	1	1	1	1	1	1	1	1	1

Table S22: Summary of the annotation of the standards mixture using XCMS-FindMain with various signal-to-noise thresholds (sn1-sn24), and prefilters either enabled and set to their default values or entirely disabled (nf).

Correctly annotated analytes are represented by a (1), while (0) signifies that the compound was either not detected, not annotated or incorrectly annotated.

ID	RT (min)	SN24	SN12	SN6	SN3	SN1	SN24-NF	SN12-NF	SN6-NF	SN3-NF	SN1-NF
M1	1.12	0	0	0	0	0	1	1	1	0	0
M2	1.15	0	0	0	0	0	1	1	1	1	1
M3	1.16	0	0	0	0	0	1	1	1	1	0
M4	1.17	0	0	0	0	0	1	1	0	1	1
M5	1.2	0	0	0	0	0	0	0	0	1	1
M6	1.2	1	1	1	1	1	1	1	1	1	1
M7	1.21	1	1	1	1	1	1	1	1	1	1
M8	1.22	0	0	0	0	0	1	1	1	1	1
M9	1.25	1	1	1	1	1	1	1	1	1	1
M10	1.26	0	0	0	0	0	0	0	0	0	0
M11	1.27	0	0	0	0	0	0	1	1	1	1
M12	1.27	1	1	1	1	1	1	1	1	1	1
M13	1.3	0	0	0	0	0	1	1	1	1	1
M14	1.31	1	1	1	1	1	1	1	1	1	1
M15	1.34	0	0	0	0	0	1	1	1	1	1
M16	1.35	0	0	0	0	0	1	1	1	1	1
M17	1.38	1	1	1	1	1	1	1	1	1	1
M18	1.56	0	0	1	1	1	0	1	1	1	1
M19	1.82	0	0	0	0	0	1	1	1	1	1
M20	1.88	0	0	0	0	0	1	1	1	1	1
M21	1.98	0	0	0	0	0	0	0	0	1	1
M22	2.08	0	0	0	0	0	0	1	1	1	1
M23	2.37	0	0	0	0	0	1	1	1	1	1
M24	2.94	1	1	1	1	1	1	1	1	1	1
M25	3.33	1	1	1	1	1	1	1	1	1	1
M26	3.86	0	0	0	0	0	1	1	1	1	1
M27	4	1	1	1	1	1	1	1	1	1	1
M28	4.25	0	0	0	0	0	1	1	1	1	1
M29	4.58	1	1	1	1	1	1	1	1	1	1
M30	4.83	0	0	0	0	0	0	0	0	0	1
M31	5.34	0	0	0	0	0	1	1	1	1	1
M32	5.7	1	1	1	1	1	1	1	1	1	1
M33	5.75	1	1	1	1	1	1	1	1	1	1
M34	5.79	0	0	0	0	0	0	0	0	1	0
M35	5.93	0	0	0	0	0	0	0	1	1	1
M36	7.01	1	1	1	1	1	1	1	1	1	1

M37	7.06	0	0	0	0	0	1	1	1	1	1
M38	7.57	1	1	1	1	1	1	1	1	1	1
M39	7.59	0	0	0	0	0	0	0	0	0	1
M40	7.62	1	1	1	1	1	1	1	1	1	1
M41	7.67	1	1	1	1	1	1	1	1	1	1
M42	7.68	0	0	0	0	0	0	0	0	0	0
M43	7.93	0	0	0	0	0	1	1	1	1	1
M44	8.53	1	1	1	1	1	1	1	1	1	1
M45	9.55	1	1	1	1	1	1	1	1	1	1
M46	10.36	1	1	1	1	1	1	1	1	1	1
M47	11.38	0	0	0	0	0	1	1	1	1	1
M48	12.86	1	1	1	1	1	1	1	1	1	1
M49	14.23	0	0	0	0	0	1	1	1	1	1
M50	14.43	1	1	1	1	1	1	1	1	1	1
M51	17.36	1	1	1	1	1	1	1	1	1	1
M52	18.34	1	1	1	1	1	1	1	1	1	1

Table S23: Summary of the annotation of the standards mixture using XCMS-mzAdan with various signal-to-noise thresholds (sn1-sn24), and prefilters either enabled and set to their default values or entirely disabled (nf).

Correctly annotated analytes are represented by a (1), while (0) signifies that the compound was either not detected, not annotated or incorrectly annotated.

ID	RT (min)	SN24	SN12	SN6	SN3	SN1	SN24-NF	SN12-NF	SN6-NF	SN3-NF	SN1-NF
M1	1.12	1	1	1	1	1	1	1	1	1	1
M2	1.15	1	1	1	1	1	1	1	1	1	1
M3	1.16	1	1	1	1	1	1	1	1	1	1
M4	1.17	1	1	1	1	1	1	1	1	1	1
M5	1.2	1	1	1	1	1	1	1	1	1	1
M6	1.2	1	1	1	1	1	1	1	1	1	1
M7	1.21	1	1	1	1	1	1	1	1	1	1
M8	1.22	1	1	1	1	1	1	1	1	1	1
M9	1.25	1	1	1	1	1	1	1	1	1	1
M10	1.26	1	1	1	1	1	1	1	1	1	1
M11	1.27	1	1	1	1	1	1	1	1	1	1
M12	1.27	1	1	1	1	1	1	1	1	1	1
M13	1.3	1	1	1	1	1	1	1	1	1	1
M14	1.31	1	1	1	1	1	1	1	1	1	1
M15	1.34	1	1	1	1	1	1	1	1	1	1
M16	1.35	1	1	1	1	1	1	1	1	1	1
M17	1.38	1	1	1	1	1	1	1	1	1	1
M18	1.56	1	1	1	1	1	1	1	1	1	1
M19	1.82	1	1	1	1	1	1	1	1	1	1
M20	1.88	1	1	1	1	1	1	1	1	1	1
M21	1.98	1	1	1	1	1	1	1	1	1	1
M22	2.08	1	1	1	1	1	1	1	1	1	1
M23	2.37	1	1	1	1	1	1	1	1	1	1
M24	2.94	1	1	1	1	1	1	1	1	1	1
M25	3.33	1	1	1	1	1	1	1	1	1	1
M26	3.86	1	1	1	1	1	1	1	1	1	1
M27	4	1	1	1	1	1	1	1	1	1	1
M28	4.25	1	1	1	1	1	1	1	1	1	0
M29	4.58	1	1	1	1	1	1	1	1	1	1
M30	4.83	0	0	0	0	0	1	1	1	1	1
M31	5.34	1	1	1	1	1	1	1	1	1	1
M32	5.7	1	1	1	1	1	1	1	1	1	1
M33	5.75	1	1	1	1	1	1	1	1	1	1
M34	5.79	1	1	1	1	1	1	1	1	1	1
M35	5.93	1	1	1	1	1	1	1	1	1	1
M36	7.01	1	1	1	1	1	1	1	1	1	1

M37	7.06	1	1	1	1	1	1	1	1	1	1
M38	7.57	1	1	1	1	1	1	1	1	1	1
M39	7.59	1	1	1	1	1	1	1	1	1	1
M40	7.62	1	1	1	1	1	1	1	1	1	1
M41	7.67	1	1	1	1	1	1	1	1	1	1
M42	7.68	0	0	0	0	0	1	1	1	1	1
M43	7.93	1	1	1	1	1	1	1	1	1	1
M44	8.53	1	1	1	1	1	1	1	1	1	1
M45	9.55	1	1	1	1	1	1	1	1	1	1
M46	10.36	1	1	1	1	1	1	1	1	1	1
M47	11.38	1	1	1	1	1	1	1	1	1	1
M48	12.86	1	1	1	1	1	1	1	1	1	1
M49	14.23	1	1	1	1	1	1	1	1	1	1
M50	14.43	1	1	1	1	1	1	1	1	1	1
M51	17.36	1	1	1	1	1	1	1	1	1	1
M52	18.34	1	1	1	1	1	1	1	1	1	1

Table S24: Summary of the peak picking process of the urine sample using XCMS with various signal-to-noise thresholds (sn1-sn24), and prefilters either enabled and set to their default values or entirely disabled (nf).

Detected analytes are represented by a (1), while (0) signifies that the feature corresponding to the protonated form of the analyte was not found.

ID	RT (min)	SN24	SN12	SN6	SN3	SN1	SN24-NF	SN12-NF	SN6-NF	SN3-NF	SN1-NF
U1	1.2	1	1	1	1	1	1	1	1	1	1
U2	1.21	1	1	1	1	1	1	1	1	1	1
U3	1.27	1	1	1	1	1	1	1	1	1	1
U4	1.27	0	0	0	0	0	0	0	0	1	1
U5	1.28	1	1	1	1	1	1	1	1	1	1
U6	1.29	1	1	1	1	1	1	1	1	1	1
U7	1.31	1	1	1	1	1	1	1	1	1	1
U8	1.35	1	1	1	1	1	1	1	1	1	1
U9	1.68	0	0	0	0	0	0	0	1	1	1
U10	1.82	1	1	1	1	1	1	1	1	1	1
U11	2	0	1	1	1	1	0	1	1	1	1
U12	2.11	1	1	1	1	1	1	1	1	1	1
U13	2.2	1	1	1	1	1	1	1	1	1	1
U14	2.62	1	1	1	1	1	1	1	1	1	1
U15	2.76	1	1	1	1	1	1	1	1	1	1
U16	3.13	0	0	0	0	0	0	0	1	1	1
U17	3.74	1	1	1	1	1	1	1	1	1	1
U18	3.97	1	1	1	1	1	1	1	1	1	1
U19	4.09	1	1	1	1	1	1	1	1	1	1
U20	4.63	0	0	0	0	0	0	1	1	1	1
U21	4.72	1	1	1	1	1	1	1	1	1	1
U22	4.83	1	1	1	1	1	1	1	1	1	1
U23	5.08	0	0	0	0	0	1	1	1	1	1
U24	5.39	1	1	1	1	1	1	1	1	1	1
U25	5.42	1	1	1	1	1	1	1	1	1	1
U26	5.55	1	1	1	1	1	1	1	1	1	1
U27	6.14	1	1	1	1	1	1	1	1	1	1
U28	6.78	1	1	1	1	1	1	1	1	1	1
U29	7.09	1	1	1	1	1	1	1	1	1	1
U30	7.24	0	0	0	0	0	1	1	1	1	1
U31	7.25	1	1	1	1	1	1	1	1	1	1
U32	9.66	0	0	0	0	0	1	1	1	1	1
U33	10.59	1	1	1	1	1	1	1	1	1	1
U34	13.24	1	1	1	1	1	1	1	1	1	1
U35	16.07	0	0	0	0	0	1	1	1	1	1

Table S25: Summary of the annotation of the urine sample using XCMS-CAMERA with various signal-to-noise thresholds (sn1-sn24), and prefilters either enabled and set to their default values or entirely disabled (nf).

Correctly annotated analytes are represented by a (1), while (0) signifies that the compound was either not detected, not annotated or incorrectly annotated

ID	RT (min)	SN24	SN12	SN6	SN3	SN1	SN24-NF	SN12-NF	SN6-NF	SN3-NF	SN1-NF
U1	1.2	0	0	0	0	0	0	0	0	0	0
U2	1.21	0	0	0	0	0	1	1	1	1	1
U3	1.27	0	0	0	0	0	0	0	0	0	0
U4	1.27	0	0	0	0	0	0	0	0	0	0
U5	1.28	0	0	0	0	0	0	0	0	0	0
U6	1.29	0	0	0	0	0	0	0	0	0	0
U7	1.31	0	0	0	0	0	0	1	1	1	1
U8	1.35	1	1	1	1	1	1	1	1	1	1
U9	1.68	0	0	0	0	0	0	0	0	0	0
U10	1.82	0	0	0	0	0	0	0	0	0	0
U11	2	0	0	0	0	0	0	0	0	0	0
U12	2.11	0	0	0	0	0	0	0	0	0	0
U13	2.2	0	0	0	0	0	0	0	0	0	0
U14	2.62	1	1	1	1	1	1	1	1	1	1
U15	2.76	0	0	0	0	0	1	1	1	1	1
U16	3.13	0	0	0	0	0	0	0	0	0	0
U17	3.74	0	0	0	0	0	0	0	0	0	0
U18	3.97	0	0	0	0	0	0	0	1	1	1
U19	4.09	0	0	0	0	0	0	0	0	0	0
U20	4.63	0	0	0	0	0	0	0	0	0	0
U21	4.72	0	0	0	0	0	0	1	1	1	1
U22	4.83	1	1	1	1	1	1	1	1	1	1
U23	5.08	0	0	0	0	0	0	0	0	0	0
U24	5.39	1	1	1	1	1	1	1	1	1	1
U25	5.42	0	0	0	0	0	0	0	0	1	0
U26	5.55	0	0	0	0	0	0	1	1	1	1
U27	6.14	1	1	1	1	1	1	1	1	1	1
U28	6.78	0	0	0	0	0	0	0	1	1	1
U29	7.09	0	0	0	0	0	0	0	0	0	0
U30	7.24	0	0	0	0	0	0	0	0	0	0
U31	7.25	1	1	1	1	1	1	1	1	1	1
U32	9.66	0	0	0	0	0	0	0	0	1	1
U33	10.59	0	0	0	0	0	0	1	1	1	1
U34	13.24	0	0	0	0	0	1	1	1	1	1
U35	16.07	0	0	0	0	0	1	1	1	1	1

Table S26: Summary of the annotation of the urine sample using XCMS-CliqueMS with various signal-to-noise thresholds (sn1-sn24), and prefilters either enabled and set to their default values or entirely disabled (nf).

Correctly annotated analytes are represented by a (1), while (0) signifies that the compound was either not detected, not annotated or incorrectly annotated.

ID	RT (min)	SN24	SN12	SN6	SN3	SN1	SN24-NF	SN12-NF	SN6-NF	SN3-NF	SN1-NF
U1	1.2	0	0	0	0	0	1	1	1	1	1
U2	1.21	0	0	0	0	0	1	0	1	1	1
U3	1.27	0	0	0	0	0	0	0	1	1	1
U4	1.27	0	0	0	0	0	0	0	0	0	0
U5	1.28	0	0	0	0	0	0	0	0	0	0
U6	1.29	0	0	0	0	0	0	0	0	0	0
U7	1.31	0	0	0	0	0	0	1	1	0	1
U8	1.35	1	1	1	1	1	1	1	1	1	0
U9	1.68	0	0	0	0	0	0	0	0	0	0
U10	1.82	0	0	0	0	0	1	1	1	1	1
U11	2	0	0	0	0	0	0	0	0	0	0
U12	2.11	0	0	0	0	0	0	0	0	0	0
U13	2.2	0	0	0	0	0	0	0	0	0	0
U14	2.62	1	1	1	1	1	1	1	1	1	1
U15	2.76	0	0	0	0	0	1	1	1	1	1
U16	3.13	0	0	0	0	0	0	0	0	1	1
U17	3.74	0	0	0	0	0	0	0	0	0	0
U18	3.97	0	0	0	0	0	0	0	1	1	1
U19	4.09	0	0	0	0	0	0	0	0	1	1
U20	4.63	0	0	0	0	0	0	0	0	0	0
U21	4.72	0	0	0	0	0	0	1	1	1	1
U22	4.83	1	1	1	1	1	1	1	1	1	1
U23	5.08	0	0	0	0	0	0	0	0	0	0
U24	5.39	1	1	1	1	1	1	1	1	1	1
U25	5.42	0	0	0	0	0	0	0	0	0	0
U26	5.55	0	0	0	0	0	0	1	1	1	1
U27	6.14	1	1	1	1	1	1	1	1	1	1
U28	6.78	0	0	0	0	0	0	0	1	1	1
U29	7.09	0	0	0	0	0	0	0	0	0	0
U30	7.24	0	0	0	0	0	0	0	0	0	0
U31	7.25	1	1	1	1	1	1	1	1	1	1
U32	9.66	0	0	0	0	0	0	0	1	1	1
U33	10.59	0	0	0	0	0	0	1	1	1	1
U34	13.24	0	0	0	0	0	0	1	1	1	1
U35	16.07	0	0	0	0	0	1	1	1	1	1

Table S27: Summary of the annotation of the urine sample using XCMS-FindMain with various signal-to-noise thresholds (sn1-sn24), and prefilters either enabled and set to their default values or entirely disabled (nf).

Correctly annotated analytes are represented by a (1), while (0) signifies that the compound was either not detected, not annotated or incorrectly annotated.

ID	RT (min)	SN24	SN12	SN6	SN3	SN1	SN24-NF	SN12-NF	SN6-NF	SN3-NF	SN1-NF
U1	1.2	1	1	1	1	1	0	0	0	0	0
U2	1.21	1	1	1	1	1	1	1	1	1	1
U3	1.27	1	1	1	1	1	1	1	0	1	1
U4	1.27	0	0	0	0	0	0	0	0	0	0
U5	1.28	1	1	1	1	1	0	0	0	0	0
U6	1.29	1	1	1	1	1	1	1	0	0	0
U7	1.31	1	1	1	1	1	1	1	1	1	1
U8	1.35	1	1	1	1	1	1	1	1	1	1
U9	1.68	0	0	0	0	0	0	0	0	0	0
U10	1.82	1	1	1	1	1	1	1	1	1	1
U11	2	0	1	1	1	1	0	0	0	0	0
U12	2.11	1	1	1	1	1	1	1	1	1	1
U13	2.2	1	1	1	1	1	1	1	1	1	1
U14	2.62	1	1	1	1	1	1	1	1	1	1
U15	2.76	1	1	1	1	1	1	1	1	1	1
U16	3.13	0	0	0	0	0	0	0	0	1	1
U17	3.74	1	1	1	1	1	1	1	1	1	1
U18	3.97	1	1	1	1	1	1	0	1	1	1
U19	4.09	1	1	1	1	1	1	1	1	1	1
U20	4.63	0	0	0	0	0	0	0	0	0	0
U21	4.72	1	1	1	1	1	1	1	1	1	1
U22	4.83	1	1	1	1	1	1	1	1	1	1
U23	5.08	0	0	0	0	0	1	1	0	0	0
U24	5.39	1	1	1	1	1	1	1	1	1	1
U25	5.42	1	1	1	1	1	1	1	1	1	1
U26	5.55	1	1	1	1	1	1	1	1	1	1
U27	6.14	1	1	1	1	1	1	1	1	1	1
U28	6.78	1	1	1	1	1	1	1	1	1	1
U29	7.09	1	1	1	1	1	1	1	1	1	1
U30	7.24	0	0	0	0	0	0	0	0	0	0
U31	7.25	1	1	1	1	1	1	1	1	1	1
U32	9.66	0	0	0	0	0	1	1	1	1	1
U33	10.59	1	1	1	1	1	1	1	1	1	1
U34	13.24	1	1	1	1	1	1	1	1	1	1
U35	16.07	0	0	0	0	0	0	0	0	0	0

Table S28: Summary of the annotation of the urine sample using XCMS-mzAdan with various signal-to-noise thresholds (sn1-sn24), and prefilters either enabled and set to their default values or entirely disabled (nf).

Correctly annotated analytes are represented by a (1), while (0) signifies that the compound was either not detected, not annotated or incorrectly annotated.

ID	RT (min)	SN24	SN12	SN6	SN3	SN1	SN24-NF	SN12-NF	SN6-NF	SN3-NF	SN1-NF
U1	1.2	1	1	1	1	1	1	1	1	1	1
U2	1.21	1	1	1	1	1	1	1	1	1	1
U3	1.27	1	1	1	1	1	1	1	1	1	1
U4	1.27	0	0	0	0	0	0	0	0	1	1
U5	1.28	1	1	1	1	1	1	1	1	1	1
U6	1.29	1	1	1	1	1	1	1	1	1	1
U7	1.31	1	1	1	1	1	1	1	1	1	1
U8	1.35	1	1	1	1	1	0	0	0	0	0
U9	1.68	0	0	0	0	0	0	0	1	1	1
U10	1.82	1	1	1	1	1	1	1	1	1	1
U11	2	0	1	1	1	1	0	1	1	1	1
U12	2.11	1	1	1	1	1	1	1	1	1	1
U13	2.2	1	1	1	1	1	1	1	1	1	1
U14	2.62	1	1	1	1	1	1	1	1	1	1
U15	2.76	1	1	1	1	1	1	1	1	1	1
U16	3.13	0	0	0	0	0	0	0	1	1	1
U17	3.74	1	1	1	1	1	1	1	1	1	1
U18	3.97	1	1	1	1	1	1	1	1	1	1
U19	4.09	1	1	1	1	1	1	1	1	1	1
U20	4.63	1	0	0	0	0	0	1	1	1	1
U21	4.72	1	1	1	1	1	1	1	1	1	1
U22	4.83	1	1	1	1	1	1	1	1	1	1
U23	5.08	0	0	0	0	0	1	1	1	1	1
U24	5.39	1	1	1	1	1	1	1	1	1	1
U25	5.42	1	1	1	1	1	1	1	1	1	1
U26	5.55	1	1	1	1	1	1	1	1	1	1
U27	6.14	1	1	1	1	1	1	1	1	0	0
U28	6.78	1	1	1	1	1	1	1	1	1	1
U29	7.09	1	1	1	1	1	1	1	1	1	1
U30	7.24	0	0	0	0	0	1	1	1	1	1
U31	7.25	1	1	1	1	1	1	1	1	1	1
U32	9.66	0	0	0	0	0	1	1	1	1	1
U33	10.59	1	1	1	1	1	1	1	1	1	1
U34	13.24	1	1	1	1	1	1	1	1	1	1
U35	16.07	0	0	0	0	0	1	1	1	1	1
